# A minimal mathematical model of red blood cell homeostasis in anemia

**DOI:** 10.1371/journal.pcbi.1014111

**Published:** 2026-03-24

**Authors:** Herut Dor, Uri Alon

**Affiliations:** 1 Gray Faculty of Medical and Health Sciences, Tel Aviv University, Tel Aviv, Israel; 2 Department of Molecular Cell Biology, Weizmann Institute of Science, Rehovot, Israel; Arizona State University, UNITED STATES OF AMERICA

## Abstract

Anemias are diverse and cause significant morbidity and mortality, but their mechanisms are not fully understood. To unravel this complexity requires understanding how hemoglobin and red blood cell function is regulated, and what goes wrong in each form of anemia. Anemias present puzzling imbalance between hemoglobin (Hb) and the main regulator of red blood cells - the hormone erythropoietin (EPO). In some anemias EPO is markedly elevated relative to the degree of anemia, whereas in others it is inappropriately low. This variability suggests that the feedback between oxygen delivery and erythropoietin production involves mechanisms beyond simple oxygen sensing. We developed a minimal mechanistic model of erythropoiesis composed of four coupled differential equations representing erythroid progenitors, marrow reticulocytes, circulating RBCs, and plasma EPO. Most parameters were derived from experimental measurements and correspond to physiological quantities. Data from ~1,830 adults across 36 published studies, encompassing healthy individuals and patients with major anemia types, were used to validate the model, along with blood-donor recovery cohorts. The model reproduces the near-exponential Hb–EPO relationship observed in reference populations, the characteristic trajectory of hemoglobin recovery after blood loss, and disease-specific deviations through physiologically plausible parameter changes, without ad hoc fitting. Elevated EPO in aplastic anemia emerges from reduced erythroid mass and diminished receptor-mediated clearance; chronic kidney disease is consistent with impaired EPO synthesis and marrow suppression; anemia of chronic disease arises from reduced progenitor differentiation without requiring primary EPO failure; and shortened RBC lifespan alone does not lower steady-state EPO in hemolysis. Of potential clinical significance is that steady-state erythropoietin levels provide a noninvasive indicator of bone marrow activity, with higher levels reflecting reduced erythroid mass and diminished receptor-mediated uptake.

## Author summary

The number of red blood cells in our blood is carefully balanced by a hormone called erythropoietin, which is produced by the kidneys. When oxygen levels drop, erythropoietin rises to stimulate the bone marrow to make more red blood cells. However, in different types of anemia, this balance behaves in unexpected ways: some patients have much more erythropoietin than their hemoglobin level would suggest, while others have far less.

We developed a simple mathematical model to understand how this system works and why it fails differently in various diseases. The model describes how red blood cells mature in the bone marrow and how erythropoietin adjusts their production through feedback. Using data from thirty-six studies, we found that small changes in a few biological parameters explain the distinct hormone patterns seen in each anemia type. Clinically, our findings suggest that erythropoietin levels may serve as a useful, noninvasive indicator of bone marrow activity in health and disease.

## Introduction

Red blood cells (RBCs) deliver oxygen to tissues via hemoglobin, which binds oxygen in the lungs and releases it in peripheral capillaries. Because tissue injury follows oxygen deprivation—within minutes in the brain and heart and hours in peripheral tissues—precise regulation of RBC production is essential. RBCs normally comprise 40–54% of blood volume [1], and deviations in either direction can have pathological consequences: deficiency impairs tissue oxygenation, whereas excess increases blood viscosity and the risk of thrombosis in the brain, heart, and gastrointestinal circulation [2].

The process regulating RBC mass, **erythropoiesis**, therefore functions as a finely tuned homeostatic system—stimulating red cell production during hypoxia and restraining it under normoxic conditions. Erythropoiesis proceeds through multiple stages, beginning with hematopoietic stem cells (HSCs) and culminating in mature erythrocytes. A key regulatory checkpoint occurs at the colony-forming unit–erythroid (CFU-E) stage, where progenitor cells can self-renew, differentiate, or undergo apoptosis. The hormone **erythropoietin (EPO)** serves as the principal dominant survival signal at this stage, preventing apoptosis of CFU-E cells and enabling their progression through the erythroid lineage [3,4].

EPO is a glycoprotein produced primarily by peritubular fibroblasts in the renal cortex [5–7]. These cells lie near the hypoxic medullary boundary, positioning them to sense minute changes in oxygen availability and rapidly expand the population of EPO-producing cells when oxygen delivery falls [8]. Under normoxia, low basal EPO levels (5–30 mU/mL) sustain steady-state erythropoiesis [9], whereas reductions in tissue oxygen tension (e.g., hypoxia, hemorrhage, or hemolysis) can trigger a more than a hundredfold increase in EPO, leading to as much as an eight-fold rise in RBC production [10].

This response is mediated by the **hypoxia-inducible factor (HIF)** pathway, the master regulator of oxygen-dependent gene expression. During hypoxia, stabilized HIF-α subunits dimerize with HIF-β and activate transcription of EPO and other hypoxia-responsive genes, generating a steep, nonlinear relationship between oxygen tension and EPO output. The resulting increase in circulating EPO stimulates erythroid progenitors in the bone marrow by binding to the homodimeric EPO receptor (EPOR) on CFU-E cells and proerythroblasts [11,12], triggering intracellular pathways that promote proliferation and inhibit apoptosis [4,13].

Together, these mechanisms constitute a **negative feedback loop**: a drop in RBC mass reduces tissue oxygenation, activating renal EPO production, which restores erythropoiesis and oxygen delivery. Conversely, RBC excess suppresses EPO synthesis, limiting further production. EPO is cleared rapidly from plasma through receptor-mediated endocytosis and lysosomal degradation [14–16], with a half-life of only a few hours.

Anemia represents one of the most prevalent disruptions of the erythropoietic system, affecting an estimated 28.2% of the global population [17]. Despite EPO being the principal regulator of red blood cell production, circulating EPO levels show remarkable variability for a given hemoglobin concentration. This might explain why EPO measurement is not routinely used for diagnosing anemia, guiding therapy selection, or monitoring treatment response. The high variability arises from both biological and methodological sources. EPO secretion displays diurnal variation [18] and reported values reflect the use of diverse assay methodologies with differing sensitivities and specificities. This variability contrasts with other endocrine systems governed by negative feedback loops—such as the thyroid axis, where thyroid-stimulating hormone (TSH) reliably reflects thyroid hormone status [19,20] and suggests that EPO regulation involves complex dynamics beyond simple oxygen feedback. Accordingly, in the present work we do not interpret individual EPO measurements as precise physiological setpoints. Instead, despite substantial variability in individual measurements, systematic deviations from the physiological EPO–hemoglobin relationship define reproducible, disease-specific patterns that can inform context-dependent interpretation of EPO levels at both the population and individual level and form the basis of the model’s interpretation.

Mathematical modeling has become a valuable tool for investigating and quantifying the regulatory mechanisms underlying physiological processes and can help to capture its essential feedback loops and cell-population dynamics into tractable equations [21–25]. Although erythropoiesis is well characterized biologically, most existing mathematical models remain limited in scope or applicability. Many focus on specific subsystems—such as intracellular signaling, progenitor proliferation, or iron metabolism—resulting in detailed models composed of dozens of coupled equations and large parameter sets [16,26–31]. These models often rely on parameter values that are estimated or fitted post hoc, rather than grounded in experimentally measured data, limiting their interpretability, generalizability, and biological transparency.

A more precise understanding of EPO dynamics could enhance diagnostic accuracy, enable earlier assessment of therapeutic efficacy—since changes in EPO levels have been observed to precede corresponding changes in hemoglobin, both during recovery from anemia [9,32] and following marrow-suppressive insults [33]- and support individualized dosing of exogenous EPO based on each patient’s EPO level.

Here we present a deliberately simplified, physiologically grounded model of erythropoiesis that integrates erythroid progenitor, reticulocyte, and RBC dynamics with EPO regulation. All but two parameters correspond to a measurable physiological quantity derived from experimental data rather than post hoc fitting, enhancing transparency and reproducibility. Despite its simplicity, the model captures key clinical behaviors of the EPO–hemoglobin system across health and multiple anemia types, providing a framework for testing mechanistic hypotheses, interpreting clinical observations, and identifying potential therapeutic interventions.

To construct and evaluate the model, we assembled a dataset of published EPO and hemoglobin values in healthy individuals and patients with the major anemia subtypes. While we are able to test the model’s dynamic aspects against recovery from blood loss, future work remains in validating it against other erythropoietic dynamics, such as recovery from chemotherapy.

## Results

### Classification of anemias by erythropoietin level

Anemia is defined as a hemoglobin level below 13 g/dL in males or 12.5 g/dL in females.

Several classification schemes exist, but a particularly useful one is based on bone marrow proliferative activity. In *hypoproliferative anemias*, the bone marrow fails to generate an adequate number of red blood cells (RBCs), whereas in *hyperproliferative anemias*, the marrow responds appropriately to the low hemoglobin by increasing RBC production, but peripheral losses, such as hemorrhage or hemolysis, cause the anemia.

**Fig 1:**
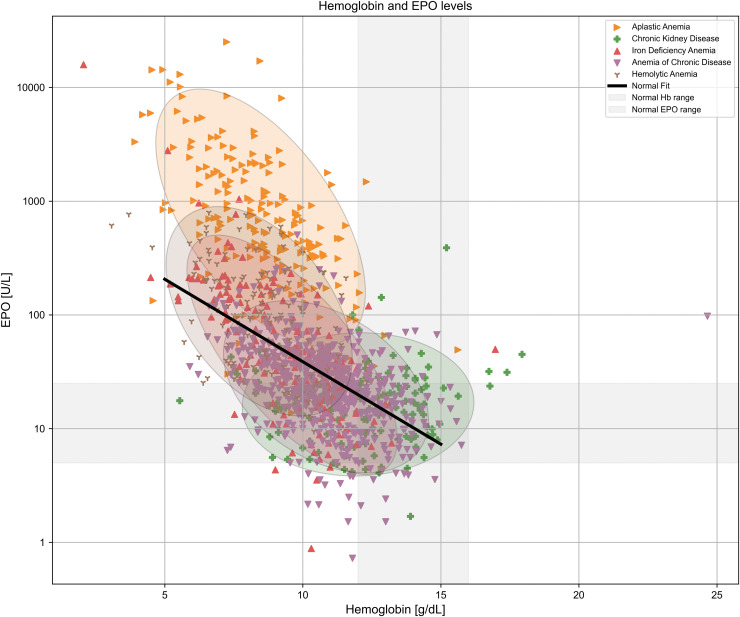
Distinct EPO–hemoglobin feedback patterns characterize major anemia subtypes. Relationship between serum erythropoietin (EPO) levels and hemoglobin concentrations across clinical anemia states. Each point represents an individual measurement extracted from published studies (sources listed in Tables 2–7). The black line indicates the baseline log-linear EPO–hemoglobin relationship (“Normal fit”), derived from reference populations defined in the original studies, which typically included healthy individuals and patients with iron deficiency anemia, as described in the section The physiological EPO–hemoglobin relationship. The gray shaded areas represent the physiological ranges of hemoglobin and EPO.

A complementary classification, central to this work, is based on circulating erythropoietin (EPO) levels [8]. Different types of anemia disrupt the EPO-hemoglobin feedback loop in characteristic ways, such that for a given hemoglobin concentration, the corresponding EPO level may deviate from what is physiologically expected (Fig 1). A summary of clinical conditions and their typical EPO deviations is presented in Table 1. Detailed analyses of each anemia subtype are presented in the subsequent sections.

**Table 1:** Classification of anemias by deviation of erythropoietin levels from expected values.

EPO is higher than expected	EPO as expected	EPO is lower than expected
Hypoplastic & aplastic anemia (AA)	Healthy population	Anemia of chronic disease (ACD)
Cytotoxic therapy [33,39]	Iron deficiency anemia (IDA)	Anemia secondary to CKD
	Hemolytic anemia (HA)	

**Table 2:** Summary of published reference data on hemoglobin and erythropoietin levels used to define the physiological EPO–hemoglobin relationship.

Study	N	Hemoglobin (g/dL)	EPO (U/L)	Method
Artunc et al. [44]	140	11.9±3.74	40.14[4.3, 51.8]	ELISA
Beguin, Yves, et al. [35] *	31	15.3±1.2	14.1	RIA
Bergamaschi, Gaetano, et al. [45]	34	9.05±1.8	259[30.8, 120.4]	ELISA
Erslev, A. J., et al. [43]	33	14.42±0.95	6.5[2.8, 8.5]	bioassay
Schrezenmeier et al. [54]	24	13.62±1.5	10.3[8.2, 12.7]	ELISA
Schreiber, Stefan, et al. [55]	42	13.2±2.3	73.37[11.8, 110.3]	RIA
Wallner, S. F., et al. [56]	19	15.25±1.1	88.47[8.6, 30.2]	bioassay

The table summarizes the reported number of participants (N), hemoglobin levels (mean ± standard deviation, g/dL), EPO levels (mean and interquartile range [25%, 75%], U/L), and assay methods used (RIA, ELISA, or bioassay) in each study. The reference population typically consisted of healthy individuals or subjects without known hematologic disorders.

*This study reported only summary statistics and did not provide the raw data; therefore, it is included in the table but not in the figure below.

**Table 3:** Summary of published data on hemoglobin and erythropoietin levels in aplastic anemia.

Study	N	Hemoglobin (g/dL)	EPO (U/L)	Method
Beguin, Yves, et al. [35] *	11	11.3±3	88.9	RIA
Das, RE Gaines, et al. [57]	41	9.1±2.1	1206[268.7, 1011.3]	RIA
Cazzola, Mario, et al. [9]	27	8.3±1.5	1614[482, 2131.3]	RIA
Erslev AJ et al. [58]	20	7.2±2	3671[1067.7, 4031.1]	Bioassay
Schrezenmeier et al. [54]	69	8.03±1.9	2359[316.23, 2187.8]	ELISA
Fattizzo, Bruno, et al. [48]	32	9.1±1.5	658[277.3, 776.9]	ELISA+
Jelkmann et al. [59]	22	7.94±1.7	2291.5[576.1, 1353.3]	RIA

The table summarizes the reported number of participants (N), hemoglobin levels (mean ± standard deviation, g/dL), EPO levels (mean and interquartile range [25%, 75%], U/L), and assay methods used (RIA, ELISA, or bioassay) across studies of patients with aplastic anemia. These data collectively illustrate that EPO concentrations are markedly elevated relative to hemoglobin levels, defining the characteristic deviation of aplastic anemia from the physiological EPO–hemoglobin relationship.

*This study reported only summary statistics and did not provide the raw data; therefore, it is included in the table but not in the figure below.

+The authors did not explicitly state the method used; however, based on the context, it appears that an ELISA-based assay was employed.

**Table 4:** Published data on hemoglobin and erythropoietin levels in anemia secondary to chronic kidney disease.

Study	N	Hb (g/dL)	EPO (U/L)	Method
Beguin, Yves, et al. [35]*	15	7.2±1.13	15	RIA
Mason-Garcia, Meredith, et al. [78] *	36	8.63±0.22	29.5	RIA
Gowanlock, Zachary et al. [79] ^*	25	9.66	25.1	ELISA
McGonigle, Richard JS, et al. [68]	60	11.4±2.5	33[19.1, 28]	RIA
Fehr et al. [72]	95	12.4±1.7	15.7[7.9, 19.15]	ELISA
Caro, Jaime, et al. [80]	14	8.4±1.5	17.8[]	bioassay
Erslev, A. J., et al. [43]	24	8.9±2.3	11.3[2.8, 17.1]	bioassay
Radtke, Heinz W., et al. [81]	88	10.95±2.3	239.8[180.8, 274]	bioassay

The table summarizes the reported number of participants (N), hemoglobin levels (mean ± standard deviation, g/dL), EPO levels (mean and interquartile range [25%, 75%], U/L), and assay methods used (RIA, ELISA, or bioassay) across studies of patients with chronic kidney disease (CKD). Together, these data show that EPO concentrations are inappropriately low relative to hemoglobin levels, defining the characteristic deviation of anemia of CKD from the physiological EPO–hemoglobin relationship.

*This study reported only summary statistics and did not provide the raw data; therefore, it is included in the table but not in the figure below.

^ The researchers reported only mean values, without providing standard deviations.

**Table 5:** Published data on hemoglobin and erythropoietin levels in hemolytic anemia.

Study	N	Hemoglobin (g/dL)	EPO (U/L)	Method
Beguin, Yves, et al. [35] *	40	10.3±2.3	66.6	RIA
Cazzola, et al. [9]	28	8±1.3	215.3[140.9, 244.1]	RIA
Fattizzo, Bruno, et al. [48] +	26	8.36±1.5	100.9[22, 65]	ELISA
Schrezenmeier et al, [54]	16	8.36±2.4	225.6[40, 358.4]	ELISA
Sherwood, Judith B., et al. [47]	14	8.25±2.2	253.7[205, 238.6]	RIA
Morgan, Anthony G., et al. [49]	31	8.8±4.4	6.9[5.9, 8.3]	Bioassay
Alexanian, Raymond. [65]	10	7.8±1	7.4[1.6, 5]	Bioassay
Theurl, Igor, et al. [84]	10	10.14±1.3	68.9[36.2, 90.4]	ELISA
Camaschella et al. [50]	30	8.4±1.2	174.8[77, 205.2]	ELISA

The table summarizes the reported number of participants (N), hemoglobin levels (mean ± standard deviation, g/dL), EPO levels (mean and interquartile range [25%, 75%], U/L), and assay methods used (RIA, ELISA, or bioassay) across studies of patients with hemolytic anemia (HA). Reported EPO concentrations vary among studies but generally fall within the physiologic range expected for the observed hemoglobin levels, indicating that the EPO response to anemia in HA is proportionate and not suppressed.

*This study reported only summary statistics and did not provide the raw data; therefore, it is included in the table but not in the figure below.

+The authors did not explicitly state the method used; however, based on the context, it appears that an ELISA-based assay was employed.

**Table 6:** Published data on hemoglobin and erythropoietin levels in anemia of chronic disease.

Study	N	Hemoglobin (g/dL)	EPO (U/L)	Method
Baer, Alan N., et al. [87]	41	10.07±1.5	38.4[18, 41]	RIA
Bergamaschi, Gaetano, et al. [45]	9	9.4±1.4	9.8[6.3, 11.9]	ELISA
Birgegård et al. [33]	13	10.94±1.4	57.6[15, 45]	RIA
Camacho, J., et al. [113]	15	10.1±1	17[10.5, 21.6]	ELISA
Cox et al. [114]^*	12	10.4±0.9	2100	bioassay
Erslev AJ et al. [58]	17	7.8±0.7	60.9[25.6, 74.6]	RIA
Gowanlock, Zachary et al. [79] ^*	31	10	16	ELISA
Hochberg et al. [115]	44	11.1±0.9	27.8[18.4, 29.1]	RIA
Kendall, R., et al. [116]	51	10.2±1.4	31.9[18.1, 38.3]	RIA
Miller, Carole B., et al. [97]	66	10.3±1.13	41.7[33.6, 48.1]	RIA
Nielsen, Ove Juul, et al. [117]	12	11.5±4.3	63[47.5, 80.2]	RIA
Pincus, Theodore, et al. [109]	17	10.16±0.87	29.3[16, 38]	immunoassay
Schett, G., et al. [106]	83	11.97±1.6	24.8[6.6, 21.7]	ELISA
Schreiber, Stefan, et al. [55]	52	12.24±1.9	39.6[16.9, 33.4]	RIA
Spivak, Jerry L., et al. [118]	63	10.37±1.6	24.2[12.5, 30.6]	RIA
Theurl, Igor, et al. [84]	9	9.75±1.66	37.4[10.4, 44.2]	ELISA
Vreugdenhil, G., et al. [119] ^*	5	10.1	15	RIA
Wood, et al. [120]	26	12.33±1.3	37.8[16.2, 61.4]	immunoassay

The table summarizes the reported number of participants (N), hemoglobin levels (mean ± standard deviation, g/dL), EPO levels (mean and interquartile range [25%, 75%], U/L), and assay methods used (RIA, ELISA, or bioassay) across studies of patients with anemia of chronic disease (ACD). Reported EPO levels vary among studies, but in most cases they are within or only modestly below the range expected for the observed hemoglobin values.

*This study reported only summary statistics and did not provide the raw data; therefore, it is included in the table but not in the figure below.

^ The researchers reported only mean values, without providing standard deviations.

**Table 7:** Published data on hemoglobin and erythropoietin levels in iron deficiency anemia.

Study	N	Hb (g/dL)	EPO (U/L)	Method
Schrezenmeier et al. [54]	23	7.9±1.9	1017.9[82.3, 380.6]	ELISA
Bergamaschi, Gaetano, et al. [45]	44	10.6±1.3	46.4[13.6, 43.9]	ELISA
Gowanlock, Zachary et al. [79] ^*	59	9.56	102.4	ELISA
Hochberg et al. [115]	17	10±1.5	63.5[20.5, 38.2]	RIA
Kendall, R., et al. [116]	54	8.45±1.8	92.5[38.5, 139.6]	RIA
Miller, Carole B., et al. [97]	21	9.8±1.3	112.7[35.4, 71.7]	RIA
Spivak, Jerry L., et al. [118]	23	10.3±2	52.3[21.9, 61.6]	RIA
Theurl, Igor, et al. [84]	32	10.6±1.2	19.6[8.4, 24.5]	ELISA
Vreugdenhil, G., et al. [119] ^*	9	9.5	60	RIA

The table summarizes the reported number of participants (N), hemoglobin levels (mean ± standard deviation, g/dL), EPO levels (mean and interquartile range [25%, 75%], U/L), and assay methods used (RIA, ELISA, or bioassay) across studies of patients with iron deficiency anemia (IDA). Reported EPO concentrations are generally consistent with the expected physiological relationship between hemoglobin and EPO.

^The researchers reported only mean values, without providing standard deviations.

*This study reported only summary statistics and did not provide the raw data; therefore, it is included in the table but not in the figure below.

Previous studies have characterized this deviation using the observed-to-predicted (O/P) ratio, which compares measured EPO levels to those expected based on hemoglobin concentration [34]. In healthy reference subjects, the O/P ratio typically falls within a 95% confidence interval ranging from 0.80 to 1.22 [35]. For each disease multiple explanations were suggested to explain the altered EPO dynamics. In the subsequent sections, we examine each proposed mechanism and test it against the predictions of our mathematical model.

This classification is not only diagnostic but also has therapeutic implications. Anemias associated with **elevated EPO levels** are generally **less likely to benefit** from exogenous EPO therapy, whereas anemias with **inappropriately low EPO levels** are **more likely to respond** to treatment [34,36]. For instance, clinical response to recombinant human erythropoietin (rhEPO) has been documented in patients with chronic kidney disease, cancer-related anemia [37], and AIDS-related anemia secondary to antiviral therapy [10] - all conditions with low EPO.

Notably, Ludwig et al. [38] found baseline EPO levels to be the only variable predictive of subsequent response to rhEPO. However, rhEPO treatment is not without risk: the most significant adverse effect is an increased incidence of thrombotic events—a risk that is often already elevated in the underlying disease itself. This underscores the importance of identifying patients most likely to benefit from EPO therapy and avoiding unnecessary treatment in others.

In this study, we use mathematical modeling to investigate how various diseases perturb erythropoiesis and the EPO–RBC feedback system. Rather than replicating disease-specific molecular details, our model abstracts common mechanistic themes to reveal generalizable features across anemia subtypes. This approach provides a framework for understanding the functional consequences of disease-driven alterations in erythropoietic regulation.

### The mathematical model

**Fig 2:**
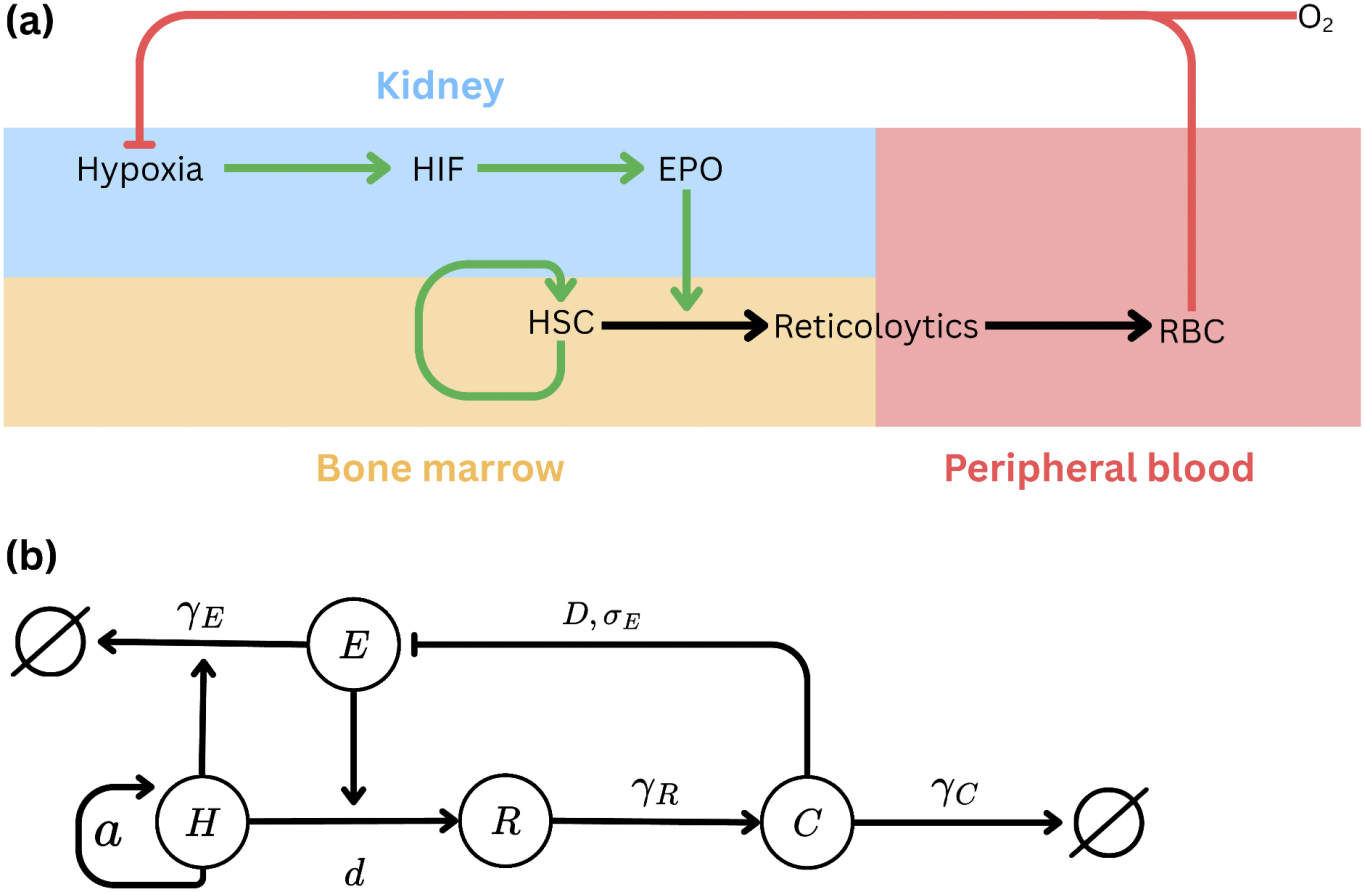
Schematic representation of the erythropoietic feedback loop and its mathematical formulation. **(a)** Conceptual overview of the physiological feedback regulating erythropoiesis. Hypoxia in the kidney activates HIF, stimulating erythropoietin (EPO) production, which promotes erythroid progenitor proliferation and differentiation in the bone marrow. Newly produced red blood cells (RBCs) restore oxygen levels, completing the feedback loop. **(b)** Corresponding structure of the mathematical model. Each node represents a compartment: erythropoietin (E), erythroid progenitors (H), reticulocytes (R), and circulating red blood cells (C). Arrows indicate production or transition rates, with parameters (a, d, γ_C_, γ_R_, D, σ_max_) corresponding to specific biological processes.

Our model is composed of 4 differential equations that describe the change of the basic components of the system over time. The structure of the erythropoietic feedback loop and its correspondence to the mathematical formulation are illustrated in Fig 2. Three of the equations describe the change in different maturation levels of the RBCs: CFU-E Hematopoietic stem cells including progenitors (denoted H(t)), Reticulocytes in the bone marrow (R(t)) and RBCs in the peripheral blood (C(t)). These three compartments were chosen as a minimal coarse-graining that captures the dominant regulatory feedback and timescales of erythropoiesis: EPO-dependent control at the progenitor stage, short-timescale buffering via reticulocyte release, and long-timescale integration through circulating red blood cell mass, while intermediate maturation stages primarily contribute finite transit times. Notably, the reticulocyte variable R(t) represents the marrow reticulocyte pool rather than the circulating reticulocytes measured in routine clinical assays; the latter can be inferred from the model variables through standard clinical estimates of release and maturation rates [14], see the supporting information S2 File. The last equation describes Erythropoietin level in the blood in units of U/L.

The basic structure of all the equations is identical: dX/dt = production term - removal term.


$$\frac{dH(t)}{dt}=a\left(E(t)\right)\cdot H(t)\cdot \left(\text{1}-\frac{H(t)}{H_{max}}\right)-d(E(t))\cdot H(t)$$ (1)

$$\frac{dR(t)}{dt}=d(E(t))\cdot H(t)-\gamma_{R}(C(t))\cdot R(t)$$ (2)

$$\frac{dC(t)}{dt}=\gamma_{R}(C(t))\cdot R(t)\;-\gamma_{c\;}\cdot C(t)$$ (3)

$$\frac{dE(t)}{dt}=\sigma_{E}\cdot exp(-\frac{C(t)}{D})-\gamma_{E}\cdot H(t)\cdot E(t)$$ (4)

Here, a(E) and d(E) are Michaelis-Menten functions representing the EPO-dependence of the proliferation rate of progenitors $a(E)\;=\frac{a_{max}\cdot E}{K_{a}+E}$, and their differentiation rate into reticulocytes $d(E)\;=\;\frac{d_{max}\cdot E}{K_{d}+E}$.

γ_R_(C) is the rate at which reticulocytes mature in the bone marrow. It is the inverse of the time reticulocytes spend in the bone marrow $\frac{\text{1}}{m_{R}\cdot C+n_{R}}$ [14].

RBC clearance is represented by a single effective degradation rate (γ_C_), corresponding to the mean RBC lifespan; this constitutes a mean-field approximation of RBC age-dependent clearance [40–42] intended for steady-state analysis. The factor $\left(\text{1}-\frac{H(t)}{H_{max}}\right)$ provides a carrying capacity to H in the bone marrow, limiting stem cell expansion as the population approaches its physiological maximum H_max_.

Notably, the erythropoietin (EPO) equation includes an endocytosis-based clearance term, since EPO is primarily removed from the circulation via receptor-mediated uptake by H.

Although in the present study γ_R_(C) and γ_C_ were perturbed only in specific disease simulations, the model formulation allows both reticulocyte maturation and red blood cell turnover to be dynamically modulated, enabling future exploration of context-dependent regulation beyond the steady-state scenarios considered here.

The value of all parameters, except d_max_, K_d_, were based on experimental measurements, although H_max_ has only been measured indirectly and with large margins of error. The value of γ_E_ was derived by combining the experimentally measured total EPO removal rate [15] with steady-state EPO and progenitor mass estimates, assuming receptor-mediated endocytosis as the dominant clearance mechanism [15,16]. The detailed calculation is shown in the supporting information S1 File.

A more complex version of the model (which we use to model dynamical data) also includes a delay, τ_R_[s], that describes the time it takes for the CFU-E cells to mature into reticulocytes $\frac{dR(t)}{dt}=d(E(t-\tau_{R}\;))\cdot H(t-\tau_{R})-\gamma_{R}(C(t))\cdot R(t)$.

### The model’s steady state

Using the dynamical equations describing the variables (H, R, C, E) of the model, we can derive its steady-state (H_st_, R_st_, C_st_, E_st_) by setting each of the equations to zero. This results in the following:


$$H_{st}=H_{max}\cdot \left[\text{1}-\frac{d(E_{st})}{a(E_{st})}\right]$$ (5)

$$R_{st}=\frac{d(E_{st})\cdot H_{st}}{\gamma_{R}(C_{st})}\;$$ (6)

$$C_{st}=\frac{\gamma_{R}(C_{st})\cdot R_{st}}{\gamma_{c\;}}=\frac{d(E_{st})\cdot H_{st}}{\gamma_{c\;}}\;$$ (7)

$$E_{st}=\frac{\sigma_{E}}{\gamma_{E}\cdot H_{st}}\cdot exp(-\frac{C_{st}}{D})$$ (8)

From the expression for EPO in steady state, Eq. (8), it is seen that the intercept of the loglinear relation between EPO and hemoglobin will be shifted if any of the parameters variables σ_E_, H_st_ or γ_E_ change. As we show below, this equation can explain the EPO levels seen in different clinical scenarios at the same hemoglobin value without the need for the complicated explanations often used in the literature. The correspondence between clinical anemia subtypes and the primary steady-state parameter shifts predicted by the model is summarized in Table 8.

**Table 8:** Mapping of clinical anemia subtypes to primary steady-state parameter shifts predicted by the erythropoiesis model.

No change	Lower σ_E_	Lower H_st_	High H_st_
Healthy population	Anemia secondary to CKD	Hypoplastic & aplastic anemia	Anemia of chronic disease (ACD)
Iron deficiency anemia (IDA)		Cytotoxic therapy	
Hemolytic anemia (HA)*			

* The current information in the literature [47–50] argues that HA shows a low level of EPO relative to the degree of anemia. As we show below, this seems unsubstantiated by the rest of the data points, as well as our model.

To simulate different clinical conditions, we modified specific model parameters as outlined in Table 9. Each scenario reflects physiologically relevant perturbations and the magnitude of the change was chosen to best fit the hemoglobin values characterized in the disease. The rationale for each modification, grounded in the underlying pathophysiology, is detailed in the following sections. Model fit was evaluated using the Wasserstein distance in units of standard deviation [51] (see Materials and Methods for details).

**Table 9:** Parameter perturbations used to simulate anemia subtypes in the erythropoiesis model.

Disease	Major pathology	Change in parameters
Hypoplastic & Aplastic Anemia (AA)	Reduced bone marrow capacity for HSCs	$H_{max}\;\to \;H_{max}/\text{7}$
Anemia secondary to CKD	Reduced EPO production	$\sigma_{\text{E}}\to \sigma_{E}/\text{1}\text{0}\text{0}$; D→ D·10; $H_{max}\;\to \;H_{max}/\text{2}$
Hemolytic Anemia (HA)	Reduced RBC lifespan	$\gamma_{C}\to \;\text{4}.\text{8}\cdot \gamma_{C}$
Anemia of chronic disease (ACD)	Reduced HSC maturation	$d_{max}\to d_{max}/\text{4}$
Iron deficiency anemia (IDA)	Reduced HSC maturation	$d_{max}\to d_{max}/\text{3}$; $a_{max}\to a_{max}/\text{3}$

We compiled a dataset of published erythropoietin (EPO) and hemoglobin (Hb) levels in adult humans. No exclusion criteria were applied during literature search, allowing inclusion of all available sources. In total, we aggregated measurements from approximately 1,830 individuals reported across 36 published studies. Reported EPO values show wide variability, reflecting both biological heterogeneity and methodological differences among studies; this variability was explicitly considered when assembling the dataset and evaluating model performance.

### Recovery following blood loss

**Fig 3:**
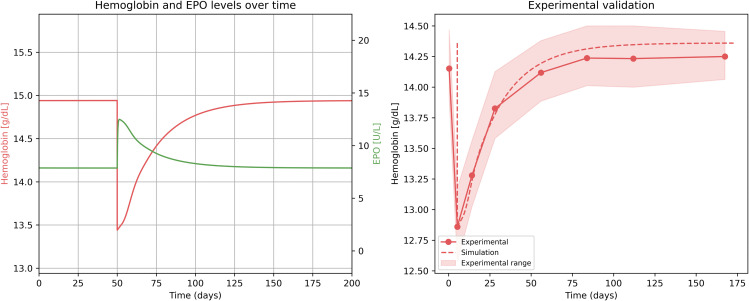
Simulated and experimental trajectories of recovery following blood loss. **(a)** Simulated hemoglobin (red) and erythropoietin (green) levels following an acute perturbation corresponding to a 0.5-L blood loss at *t* = 50 days. **(b)** Comparison between simulated hemoglobin dynamics (dashed line) and experimental data from Kiss et al. [52] (solid line, mean ± range).

We first examined the behavior of the model under an acute blood loss of 0.5 L, equivalent to the volume removed during a standard blood donation. The simulation began at steady state, and at time t = 50 days, a sudden loss of 0.5 L of blood was introduced. This perturbation resulted in a rapid decline in hemoglobin levels, followed by a sharp rise in erythropoietin (EPO) concentration (Fig 3a). The elevated EPO level stimulated increased red blood cell (RBC) production, eventually restoring hemoglobin to its baseline value within about a month.

To evaluate the accuracy of the model’s predictions, we compared the simulated hemoglobin recovery trajectory to experimental data from a study by Kiss et al.[52], which investigated hemoglobin dynamics following blood donation. We focused on the subgroup of participants with high baseline ferritin who received iron supplementation, in order to match the iron-replete conditions assumed in our simulation. Accordingly, this analysis isolates erythropoietic recovery driven primarily by EPO-mediated feedback, whereas iron limitation - which is known to contribute substantially to inter-individual variability in hemoglobin recovery after blood donation [53] - is not explicitly considered here. Using this dataset, we reconstructed the time course of hemoglobin recovery. As shown in Fig 3b, the model accurately reproduced the qualitative pattern of recovery. Quantitatively, the simulation predicted 80% hemoglobin restoration at 37.5 days, roughly matching the experimental value of 31 ± 2 days.

Next, for each anemia type, we present a summary table compiling the available data on hemoglobin and erythropoietin (EPO) levels specific to that condition. For each dataset, we calculated the mean and standard deviation of hemoglobin, and the mean and interquartile range of EPO. These results are followed by graphical comparisons between the experimental data and the corresponding model simulations, illustrating the model’s ability to reproduce observed patterns across different anemia states.

### The physiological EPO–hemoglobin relationship

The composition of the reference group varied across studies but generally included two main subpopulations: healthy individuals representing the normal hemoglobin range (i.e., 12–15 g/dL), and patients with iron deficiency anemia accounting for most of the anemic values. These two groups collectively defined the physiological spectrum of erythropoietin responses. This reference population was used to construct the baseline log-linear relationship between erythropoietin (logEPO) and hemoglobin, against which deviations in other anemic conditions were compared in the remainder of this study.

**Fig 4:**
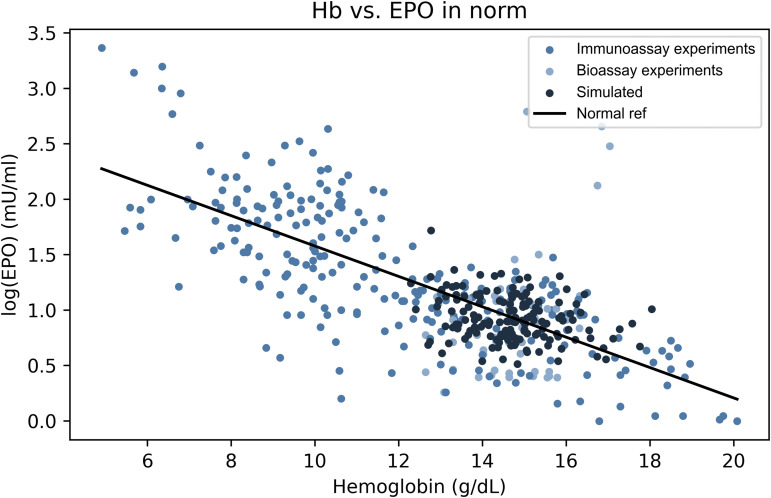
The physiological relationship between hemoglobin and erythropoietin levels. Comparison of the simulated EPO–hemoglobin relationship (dark blue points, *n* = 150) with experimental data from 292 individuals across reference populations (blue points). The regression line derived from empirical data (black line) defines the baseline log-linear relationship between log(EPO) and hemoglobin. This line serves as the physiological reference used in subsequent comparisons with pathological conditions.

Fig 4 illustrates the strong concordance between the model and empirical data across the physiological range of hemoglobin levels. The Wasserstein distance was 0.6 standard deviations (see Methods), indicating similarity between the two distributions.

### Aplastic anemia

Aplastic anemia (AA) is a form of bone marrow failure characterized by pancytopenia and a markedly hypocellular marrow. Hematopoietic stem cells (HSCs) are severely reduced or absent, often due to immune-mediated destruction triggered by factors such as radiation, drugs, or viral infections. As a result, the production of red blood cells (RBCs), white blood cells, and platelets is profoundly impaired. The anemia associated with AA is typically severe, as the bone marrow is unable to mount an adequate erythropoietic response, even under strong hypoxic stimuli.

In AA, erythropoietin (EPO) levels are significantly elevated for any given hemoglobin (Hb) concentration compared to other types of anemia [9,35,48,54,57–59]. This finding is consistent even among patients in hematologic remission [54].

Several hypotheses have been proposed to explain this exaggerated EPO response.

One possibility is that the production of inflammatory cytokines, particularly interleukin-1 (IL-1) and tumor necrosis factor-alpha (TNF-α), reduces the oxygen dependency of EPO synthesis [60]. However, this explanation appears unlikely, as diseases associated with high IL-1 levels, such as rheumatoid arthritis and chronic infections, typically show suppressed rather than elevated EPO responses. Furthermore, studies have reported low IL-1 levels in patients with AA [61].

A second proposed mechanism is EPO resistance, where impaired responsiveness of erythroid progenitors to EPO leads to compensatory elevations in circulating EPO levels [62].

A third explanation is based on reduced EPO clearance due to diminished erythroid mass. Under this hypothesis, lower erythroid activity reduces receptor-mediated EPO removal, resulting in higher plasma EPO concentrations [54]. Supporting this idea, some studies have observed an inverse correlation between EPO levels and markers of erythroid activity, such as serum transferrin receptor (sTfR) concentrations [9]. However, other studies did not confirm this association [63–65], suggesting that the mechanisms underlying EPO elevation in AA may be multifactorial.

We model this disease by reducing the bone marrow’s maximal capacity for erythroid progenitor cells, achieved by decreasing $H_{max}$ to one-seventh of its baseline value, ${H^{\prime }}_{max}=H_{max}/\text{7}$ (see Table 9). Given the model’s coarse-grained structure, H_max_ represents an aggregate erythropoietic capacity, and a large reduction may reflect simplification rather than near-complete loss of a single biological process.

The simulation results aligned with the experimental observations, yielding a Wasserstein distance of 0.67 standard deviations (whereas the distance between the disease model and the healthy population was 8.5 standard deviations, indicating a substantially poorer match).

These findings support the hypothesis that reduced erythroid activity is the dominant contributor to the elevated EPO levels observed in aplastic anemia. While alternative mechanisms, such as cytokine-mediated modulation or EPO resistance, may contribute to some extent, modeling them as the primary effect—via changes in EPO production parameters such as $\sigma_{E}$ and D, or in parameters associated with EPO clearance—results in either substantially lower EPO levels for a given hemoglobin range or unrealistically high hemoglobin values for the observed EPO distribution, depending on the direction of the perturbation. This supports modeling aplastic anemia primarily through a reduction in erythroid mass ($H_{\text{max}}$), which indirectly decreases receptor-mediated EPO clearance while maintaining low hemoglobin.

Moreover, these findings support a role of receptor-mediated endocytosis by erythroid precursors as a mechanism contributing to EPO clearance.

### Anemia secondary to chronic kidney disease

Anemia secondary to chronic kidney disease (CKD) is a common early complication, typically presenting as normochromic, normocytic anemia with hypoproliferative bone marrow. It usually emerges when the glomerular filtration rate (GFR) falls below 60 mL/min/1.73 m², with severity correlating with the degree of renal impairment [44,66]. In CKD patients, anemia significantly reduces quality of life and is associated with increased cardiovascular and all-cause mortality [67].

The cause of anemia in CKD is multifactorial and includes EPO deficiency, iron deficiency from chronic blood loss or reduced gastrointestinal absorption, impaired utilization of iron stores due to elevated hepcidin levels, systemic inflammation, suppression of bone marrow by uremic toxins [68,69], shortened red blood cell lifespan [70], and deficiencies in vitamin B12 or folate [71].

Several studies have shown that while erythropoietin (EPO) levels in CKD patients are elevated compared to healthy individuals, they remain inappropriately low relative to the degree of anemia [44,66]. In advanced CKD, EPO production becomes largely independent of hemoglobin levels [72]. The mechanisms underlying EPO deficiency in CKD remain incompletely understood.

Several hypotheses have been proposed, including direct injury and fibrosis of the peritubular fibroblasts responsible for EPO production; a rightward shift in oxygen-sensing sensitivity due to chronically reduced renal perfusion [73]; accumulation of uremic toxins; and urinary loss of EPO caused by impaired tubular reabsorption. While the latter has been demonstrated in nephrotic syndrome [74], it is unlikely to represent a major contributor in CKD, as EPO clearance by the kidney is considered minor [75], and EPO secretion rates have been shown to be similar in uremic and healthy individuals [76].

Importantly, this defect in EPO production appears to be at least partially reversible: native kidney EPO secretion often recovers following successful kidney transplantation [77].

Experimental data comparing patients with chronic kidney disease (CKD) to healthy individuals demonstrate a reduction in both the slope and the intercept of the log(EPO) versus hemoglobin relationship [44]. In our model, these observations are captured by decreasing the parameter $\sigma_{E}$, which sets the upper limit of erythropoietin production (affecting the intercept), and increasing D, which modulates the steepness of the EPO response to hemoglobin changes (affecting the slope). To capture these shifts, we applied heuristic adjustments—reducing $\sigma_{E}$ by a factor of 100 and increasing D tenfold, ${\sigma^{\prime }}_{E}=\sigma_{E}/\text{1}\text{0}\text{0};\;D^{\prime }=D\cdot \text{1}\text{0}$. These values were chosen empirically to align with the range and shape of the experimental data. While not directly derived from biological measurements, they provided a reasonable approximation of the observed EPO-Hb relationship in CKD. A sensitivity analysis around the CKD parameter regime showed that the characteristic EPO–hemoglobin phenotype is reproduced only within a narrow range of parameter values (approximately ±20%), indicating that the data tightly constrain the parameters to a specific physiological regime rather than allowing the CKD pattern to emerge from arbitrary parameter choices.

However, these changes alone were insufficient to reproduce the full extent of anemia observed in CKD patients. To improve the model’s fit, we incorporated an additional mechanism—bone marrow suppression, likely reflecting the inhibitory effects of uremic toxins on erythropoiesis. This was implemented by reducing $H_{max}$, the maximal capacity for erythroid progenitor proliferation, to 50% of its baseline value, ${H^{\prime }}_{max}=H_{max}/\text{2}$ (see Table 9).

As shown in Fig 6, the model accurately captures the distribution of hemoglobin and erythropoietin (EPO) levels in the population and reproduces the near independence between EPO concentration and hemoglobin. Note that data points from bioassay studies are significant outliers. The weak association between circulating erythropoietin and hemoglobin reflects a breakdown of normal EPO–hemoglobin feedback regulation in chronic kidney disease. This pattern suggests that a substantial fraction of hemoglobin variability is driven by factors acting independently of endogenous EPO levels. Plausible contributors include iron availability and utilization, inflammatory tone, degree of renal dysfunction, uremic marrow suppression, nutritional deficiencies, and altered red blood cell lifespan. From a statistical perspective, these factors would be expected to contribute to hemoglobin variability without a corresponding change in EPO, thereby weakening the observed EPO–hemoglobin association.

Consistent with this interpretation, the Wasserstein distance was 0.99 standard deviations, whereas the corresponding distance between the disease model and the healthy population was 7.8 standard deviations, indicating a substantially poorer fit.

Notably, this fit could not be achieved without incorporating the component of bone marrow suppression. This finding suggests that impaired erythropoietic capacity—rather than EPO deficiency alone—plays a significant role in the pathophysiology of anemia secondary to chronic kidney disease (CKD). Supporting this interpretation, previous studies have reported increased rates of hypocellular bone marrow in patients with advanced CKD [82], along with a higher prevalence of reductions in other hematopoietic lineages [83], indicating a broader suppression of marrow function in this population.

### Hemolytic anemia

Hemolysis refers to the premature destruction of red blood cells (RBCs). *Hemolytic anemia* develops when the rate of RBC loss exceeds the compensatory capacity of the bone marrow. The underlying causes of hemolysis may be intrinsic to the RBC—such as enzymopathies (e.g., glucose-6-phosphate dehydrogenase [G6PD] deficiency), hemoglobinopathies (e.g., thalassemia), or membranopathies (e.g., hereditary spherocytosis)—or extrinsic, including autoimmune disorders, infections, drug toxicity, and mechanical destruction (e.g., prosthetic heart valves).

EPO levels in many patients with chronic hemolytic disorders are inappropriately low relative to the degree of anemia [47–50]. Several explanations have been proposed for this. One hypothesis suggests that the robust reticulocyte response to hemolysis reverses the relative hypoxia associated with anemia, thereby reducing the stimulus for renal EPO production—a phenomenon also observed in transfused patients [48]. In addition, compensatory physiological adaptations such as increased cardiac output and expanded blood volume may enhance tissue oxygenation and suppress EPO secretion [65].

Elevated levels of inflammatory cytokines, which are often present in hemolytic states, may further impair EPO synthesis [48]. In diseases such as sickle cell anemia, renal damage may directly interfere with EPO production; this is supported by evidence linking EPO levels to creatinine clearance and by age-related differences in EPO responsiveness, with younger patients showing higher EPO levels for the same hemoglobin concentration [47,49].

Additionally, a rightward shift in the oxygen–hemoglobin dissociation curve in these patients improves oxygen delivery to tissues and hence blunt the hypoxic signal required for EPO induction [47]. Finally, in conditions such as sickle cell disease, the bone marrow may expand its erythropoietic output so effectively that less circulating EPO is required to sustain RBC production [65].

In contrast to the studies described above, several others did not find erythropoietin (EPO) levels to be lower than expected for the degree of anemia [35,65]. Some reports observed only that EPO concentrations were reduced relative to patients with aplastic anemia—a group known to exhibit markedly elevated EPO levels (see Results - Aplastic anemia) [9,54].

When the available data are aggregated, EPO levels in hemolytic anemia are generally within, or slightly above, the expected range for a given hemoglobin concentration (see Fig 7). This observation is also consistent with predictions from our model, in which a shortened red blood cell lifespan does not significantly alter steady-state EPO levels (E_st_). This result reflects steady-state behavior within a mean-lifespan framework and therefore does not capture age-dependent clearance mechanisms or transient dynamics.

To model hemolytic anemia (HA), the red blood cell (RBC) lifespan was reduced from 120 days to 25 days, consistent with reported estimates of 10–62 days for RBC survival in hemolytic anemia [85,86]. This change was implemented in the model by increasing the clearance rate parameter $\gamma_{c}$, such that ${\gamma^{\prime }}_{c}={\text{4}.\text{8}\cdot \gamma }_{c}$. Here, hemolytic anemia is analyzed under the assumption of sustained hemolysis, such that the system reaches a steady state on a timescale set by the shortened RBC lifespan.

As shown in Fig 7, the experimental data display EPO levels that are within the normal range or modestly elevated. The model simulation aligns with these observations, demonstrating good agreement between predicted and observed EPO responses. Note that data points from bioassay studies are significant outliers. The Wasserstein distance was 1 standard deviations, in contrast to 7.3 standard deviations obtained when comparing the disease model with the healthy population, underscoring the markedly inferior fit of the latter. The close alignment of the data with the reference EPO–hemoglobin relationship indicates that the core feedback structure is largely preserved under sustained hemolysis. The remaining scatter around the reference line is therefore more plausibly attributed to heterogeneity in hemolysis severity and chronicity (effective red blood cell lifespan), iron status and reticulocyte response, comorbid inflammation, and between-study or assay-related measurement variability. From a statistical perspective, these factors are expected to introduce dispersion around an intact feedback relationship rather than producing a systematic breakdown of EPO–hemoglobin coupling.

### Anemia of chronic disease

Anemia of chronic disease (ACD), also known as anemia of inflammation, is a hypoproliferative anemia commonly associated with chronic infections, autoimmune disorders, inflammatory conditions, and non-hematologic malignancies. It typically presents as a normocytic, normochromic anemia with a reduced erythropoietic response. In our analysis, we focused on patients with non-hematologic cancers who were not receiving chemotherapy, to exclude anemia mechanisms specific to hematologic malignancies or cytotoxic treatment.

In many cases, circulating erythropoietin (EPO) levels are inappropriately low relative to the degree of anemia [87]. However, other studies have reported EPO concentrations that appear appropriate for the level of hemoglobin [33,58], likely reflecting heterogeneity among chronic disease populations. Taken together, these reports highlight substantial heterogeneity in circulating EPO levels in anemia of chronic disease, while still supporting population-level inference about systematic deviations from the physiological EPO–hemoglobin relationship.

The pathogenesis of ACD is multifactorial and not yet fully elucidated. Proposed mechanisms include a mildly reduced red blood cell lifespan [88], functional iron deficiency due to hepcidin-induced sequestration of iron within the reticuloendothelial system [89–91], and direct suppression of erythropoiesis by inflammatory cytokines, which impair the survival and differentiation of erythroid progenitors [92,93]. Additional evidence indicates that interferon-γ and interleukin-1 may reduce the expression of EPO receptors on erythroid progenitor cells [94,95], though in vitro research shows normal response of erythroid progenitor cell to EPO, concurrent with low EPO [96].

Cytokine-mediated suppression of renal EPO synthesis is a widely accepted explanation for this phenomenon. Reduced EPO production has been described in chronic inflammatory states such as rheumatoid arthritis [92], malignancy [97,98], congestive heart failure [99], and HIV/AIDS [100]. In vitro experiments have shown that pro-inflammatory cytokines, including IL-1, TNF-α, TGF-β, and IFN-γ, directly inhibit EPO gene expression [15,101,102].

Histological studies in lupus nephritis further support this view, revealing inflammatory infiltration of the renal interstitium, where EPO-producing fibroblasts reside [103]. One proposed mechanism suggests that chronic inflammation induces a phenotypic transformation of these fibroblasts into myofibroblasts, rendering them incapable of producing EPO [104]. EPO deficiency may, in turn, contribute to elevated hepcidin levels, exacerbating functional iron restriction [105]. In some cases, anti-EPO autoantibodies have also been identified, particularly in systemic lupus erythematosus, and are associated with lower EPO levels [106].

An interesting hypothesis raised by [107] suggests that ACD may involve a pre-anemic state characterized by elevated EPO levels required to maintain hemoglobin within the normal range. In their population-based study, they observed an association between elevated inflammatory markers (CRP, IL-6, IL-1, TNF-α) and increased EPO concentrations in individuals without anemia, whereas in those with anemia, greater inflammation was paradoxically associated with lower EPO levels. This implies that inflammation alters the EPO response in a stage-dependent manner. Supporting this concept, studies have shown that resistance to recombinant human EPO (rHuEPO) therapy correlates with higher levels of pro-inflammatory cytokines [108].

Further supporting the role of insufficient EPO in ACD is the demonstrated benefit of EPO therapy in various chronic disease states. Clinical studies have shown improvement in hemoglobin levels following rHuEPO administration in patients with rheumatoid arthritis [109,110], HIV infection receiving zidovudine [111], inflammatory bowel disease [55], and cancer [37,112].

To simulate anemia of chronic disease (ACD), we modeled the effects of impaired erythropoiesis by reducing the differentiation rate from hematopoietic stem cells to red blood cells by a factor of four, $\overset{\prime }{\underset{max}{d}}=d_{max}/\text{4}$ (see Table 9). This adjustment reflects the combined impact of inflammatory cytokines and functional iron deficiency, both of which are known to suppress erythroid maturation. Importantly, erythropoietin (EPO) production was unchanged in the simulation.

As depicted in Fig 8, the model reproduces the joint distribution of hemoglobin and erythropoietin (EPO) levels with high fidelity, yielding a Wasserstein distance of 0.53 standard deviations. In contrast, comparison of the disease model with the healthy population distance of 7.3 standard deviations, reflecting a substantially inferior fit.

Notably, the simulation did not impose a blunted EPO response, indicating that reduced EPO levels are not necessary to generate this pattern of anemia and may instead represent a downstream consequence of other disease mechanisms rather than a primary cause.

### Iron deficiency anemia

Iron deficiency anemia (IDA) arises from a deficiency in iron, which is essential for hemoglobin synthesis and hence to RBC production. IDA is characterized by hypo-proliferative microcytic anemia.

IDA is often referred to as simple anemia and in many cases it is used as a benchmark for the expected EPO level in anemia states. Because the expectation is that IDA does not affect the production or removal mechanism of EPO, patients with IDA can be used to extrapolate the linear trend line that relates hemoglobin and EPO for healthy levels of hemoglobin to levels of hemoglobin associated with disease.

A surge in iron receptors (TfR1) occurs when BFU-E cells transform to CFU-E cells and peak at the pro-erythroblasts stage [121]. Several researches have shown decrease in CFU-E cell count in vitro when iron is decreased [122,123]. Hence, we choose to simulate IDA by decreasing both $a_{max},\;d_{max}$ by a factor of 3 $\left({{a^{\prime }}_{max}\;=\;a}_{max}/\text{3};\;{d^{\prime }}_{max}\;=d_{max}/\text{3}\right)$.

Fig 9 illustrates close agreement between the experimental observations and the model simulations. The Wasserstein distance was 0.68 standard deviations, whereas comparison of the disease model with the healthy population yielded a value of 7, reflecting a markedly inferior fit.

**Fig 5:**
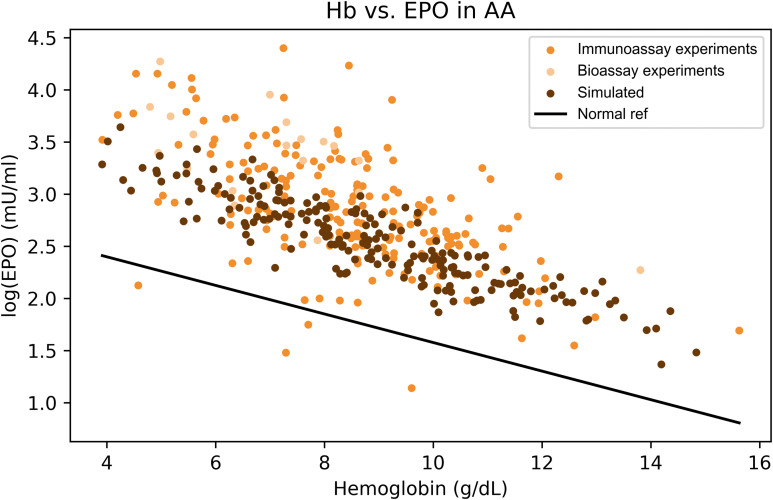
The model reproduces the elevated erythropoietin response characteristic of aplastic anemia. Comparison of simulated data (dark points, *n* = 200) with experimental measurements of log(EPO) versus hemoglobin from 211 patients with aplastic anemia (orange points). The solid black line represents the reference log-linear EPO–hemoglobin relationship derived from non-anemic populations. Both the empirical and simulated data show EPO levels that are markedly higher than expected for a given hemoglobin concentration, consistent with reduced erythroid mass and impaired EPO clearance in aplastic anemia.

**Fig 6:**
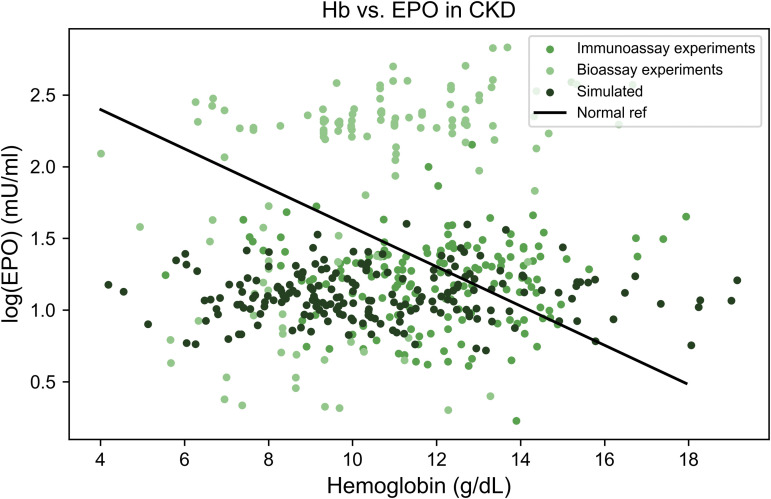
The model reproduces the blunted erythropoietin response characteristic of chronic kidney disease. Comparison of experimental data (green points, *n* = 317) and model simulations (dark green points, *n* = 200) for log(EPO) versus hemoglobin levels in chronic kidney disease (CKD). The solid black line represents the reference EPO–hemoglobin relationship derived from non-anemic populations. Both experimental and simulated data show a downward shift and flattening of the curve, consistent with reduced EPO production and bone marrow suppression in CKD.

**Fig 7:**
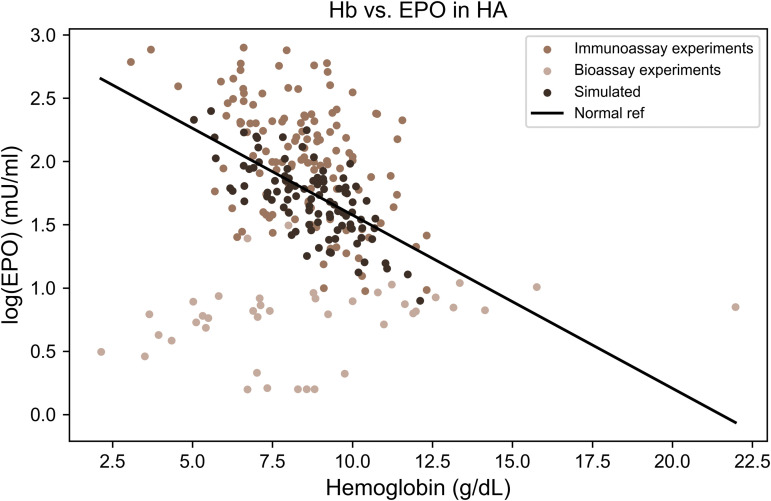
Erythropoietin levels increase proportionally to anemia severity in hemolytic anemia. Comparison of experimental data (light brown points, *n* = 165) and model simulations (dark brown points, *n* = 100) for log(EPO) versus hemoglobin levels in hemolytic anemia (HA). The solid black line represents the reference EPO–hemoglobin relationship derived from non-anemic populations. Both empirical and simulated data show EPO levels that increase in proportion to the severity of anemia, consistent with a physiologically appropriate feedback response despite accelerated red blood cell turnover.

**Fig 8:**
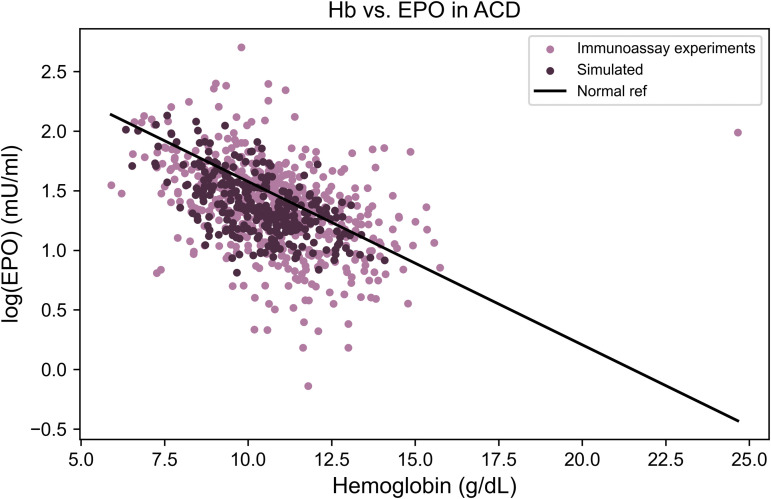
Impaired erythroid differentiation accounts for the EPO–hemoglobin relationship in anemia of chronic disease. Comparison of experimental data (purple points, *n* = 518) and model simulations (dark purple points, *n* = 300) for log(EPO) versus hemoglobin levels in anemia of chronic disease (ACD). The solid black line represents the reference EPO–hemoglobin relationship derived from non-anemic populations. Both experimental and simulated data show EPO levels that are near or slightly below the expected physiological range for a given hemoglobin concentration. The model reproduces this distribution without assuming reduced EPO production, indicating that impaired erythroid differentiation alone can explain the observed pattern.

**Fig 9:**
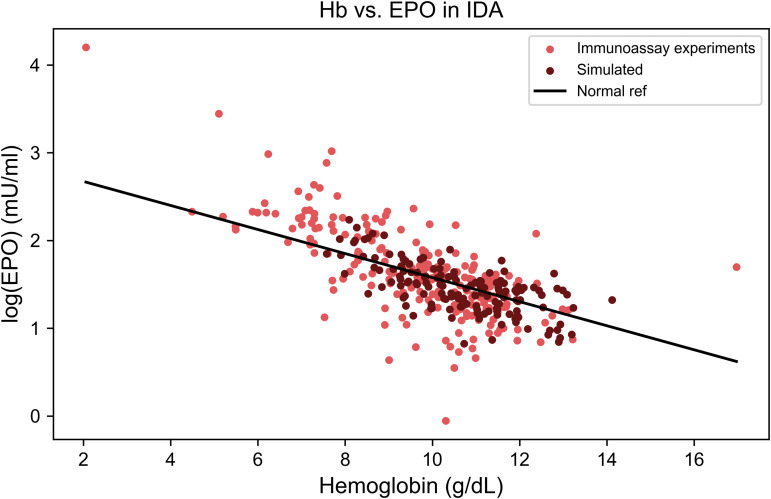
The model reproduces the physiological erythropoietin response in iron deficiency anemia. Comparison of experimental data (light red points, *n* = 214) and model simulations (dark red points, *n* = 150) for log(EPO) versus hemoglobin levels in iron deficiency anemia (IDA). The solid black line represents the reference EPO–hemoglobin relationship derived from non-anemic populations. Both experimental and simulated data align closely with this relationship, indicating that EPO production is appropriately regulated despite reduced erythroid proliferation and differentiation caused by iron limitation.

Patients with iron-deficiency anemia align closely with the expected physiological relationship between hemoglobin and erythropoietin (EPO), falling along the reference loglinear relationship derived from healthy individuals.

### Potential target for intervention

The model can also be used to explore potential therapeutic strategies for different anemia types by varying parameters other than those specifically modified to simulate each condition. The current therapeutic principle for most anemia types is to treat the underlying cause. While this is straightforward in certain cases—such as iron supplementation in iron-deficiency anemia—it is considerably more challenging, or even impossible, in others, such as anemia of chronic disease (where no effective treatment exists for the primary illness) or hereditary hemolytic anemia.

We first outline the general therapeutic principles that emerge from the model and then discuss how they manifest in each anemia subtype. The parameters examined as possible therapeutic targets include: (1) H_max_, representing the carrying capacity of erythroid progenitors. Increasing this parameter would correspond to expansion of the erythropoietic compartment, a compensatory mechanism that develops under certain pathological conditions; (2) parameters that elevate EPO levels (E_max_, D, γ_E_), reflecting the effect of exogenous EPO administration; (3) parameters influencing progenitor proliferation (a_max_, k_a_); and (4) parameters affecting differentiation from progenitors to reticulocytes (d_max_, k_d_).

At present, no pharmacologic agents are known to selectively enhance erythroid proliferation or differentiation in a manner analogous to granulocyte colony-stimulating factor (G-CSF) in the myeloid lineage. Changes in γ_c_ (red blood cell lifespan), while physiologically important, were not examined because they are difficult to selectively and predictably modulate as a therapeutic control parameter. It is noteworthy that while a mild increase in d_max_ (progenitor differentiation rate) may enhance erythroid output, a larger increase can have the opposite effect by depleting the progenitor pool.

The model identifies three major therapeutic principles. First, exogenous EPO is expected to be beneficial when steady-state EPO levels are not already elevated relative to hemoglobin levels. Second, increasing H_max_—the carrying capacity—would be beneficial only when the steady-state progenitor count approaches this upper limit. Third, increasing the proliferation rate of progenitor cells (via a_max_) may be effective if the progenitor population is well below its maximal carrying capacity.

In hemolytic anemia, one of the body’s known compensatory mechanisms is the expansion of erythropoietic tissue into additional bones (e.g., skull) or the development of extramedullary hematopoiesis in organs such as the spleen [124]. The model reproduces this effect: increasing H_max_ raises the steady-state hemoglobin level. Similarly, elevating steady-state EPO levels also increases hemoglobin. This finding aligns with clinical observations that EPO therapy can be effective in specific subtypes of hemolytic anemia, such as autoimmune hemolytic anemia (AIHA) [48], where endogenous EPO levels are lower than expected for the degree of anemia.

In aplastic anemia, EPO levels are already elevated, so exogenous EPO is unlikely to help. Because the number of progenitor cells is markedly reduced, increasing a_max_ (proliferation rate) and d_max_ (differentiation rate) is expected to improve the condition.

In anemia of chronic disease, an increase in H_max_ could be beneficial, although such an effect is unlikely to occur physiologically. Unlike hemolytic anemia, anemia of chronic disease tends to be mild and occurs mainly in adults, in whom bone marrow expansion is limited.

In anemia secondary to chronic kidney disease (CKD), erythropoietin production is impaired, resulting in inappropriately low circulating EPO levels for the degree of anemia. Consequently, exogenous EPO administration is therapeutically effective. In the model, increasing a_max_ (progenitor proliferation rate) or d_max_ (differentiation rate) enhances erythroid output by amplifying the number of progenitor cells that proliferate or differentiate per unit of available EPO, thereby partially compensating for the hormonal deficiency.

Our current model does not include a representation of iron availability and therefore cannot simulate therapeutic strategies for iron-deficiency anemia. One possible extension would be to constrain the terms H(t)d(E) and H(t)a(E)(1 – H/H_max_) by introducing an iron factor: min[H(t)d(E), iron factor]. This would limit differentiation and proliferation according to iron supply. Under such a framework, increasing H_max_ or steady-state EPO would not alleviate anemia, as the bottleneck would lie downstream.

## Discussion

In this study, we introduced a simplified mathematical model capturing three key stages of red blood cell (RBC) production along with erythropoietin (EPO) dynamics. This model offers several strengths. First, the parameters were defined a priori based on experimental measurements rather than post hoc fitting, enhancing the model’s biological transparency. Second, the model was validated against a comprehensive dataset aggregated from 36 studies across diverse populations and measurement techniques, providing strong empirical support and making it a potential data resource for future modeling efforts. Third, rather than focusing on a single disease, we evaluated the model across multiple anemia subtypes, enabling it to reveal general physiological principles underlying erythropoiesis.

Using experimentally derived parameter values with biologically plausible variation, we demonstrated that the model reproduces observed hemoglobin and EPO levels in both healthy adults and patients with five major types of anemia. Importantly, the model also reproduced compensatory responses observed in vivo—such as expansion of erythropoietic activity into additional bones in hemolytic anemia—and identified parameter perturbations that may serve as potential therapeutic targets. This agreement between simulations and experimental data allows the model to be used to examine unresolved questions—particularly the longstanding clinical observation that EPO levels often differ substantially between diseases, even at similar hemoglobin concentrations.

By analyzing the system’s steady-state solutions, the model identifies distinct shifts in key regulatory parameters that explain these deviations. In **aplastic anemia**, elevated EPO levels can be attributed to reduced EPO uptake by erythroid progenitors due to their reduced numbers, resulting in lower EPO clearance. In **chronic kidney disease (CKD)**, the model indicates that blunted EPO production and EPO deficiency are necessary to reproduce the observed EPO suppression; however, additional bone marrow suppression—likely due to uremic toxins—is required to fully explain the clinical data. In **hemolytic anemia**, despite some reports of inappropriately low EPO, the aggregate data and simulation results do not support this interpretation. The model suggests that reducing RBC lifespan alone does not significantly impact steady-state EPO levels, suggesting a need to re-evaluate prevailing assumptions about this condition’s pathophysiology. In **anemia of chronic disease (ACD)**, the model reproduces characteristic features of the anemia without requiring a blunted EPO response. Our findings suggest that low EPO levels may be a *consequence* of decreased CFU-E differentiation, rather than a primary cause. In **iron deficiency anemia (IDA)**, reproducing the clinical data required reducing both stem cell proliferation and erythroid cell division rates, supporting the hypothesis that iron availability influences not only differentiation but also proliferation of erythroid precursors. The model’s steady state is highly robust to parameter changes within biologically relevant parameter ranges and does not exhibit oscillatory behavior.

A mechanistic insight emerging from the model is the regulatory role of receptor-mediated endocytosis in the clearance of circulating erythropoietin (EPO). Because EPO is primarily removed through binding to erythroid precursors, its plasma concentration is strongly influenced by bone marrow erythropoietic activity. This relationship enables EPO levels—when interpreted relative to hemoglobin—to serve as a potential indicator of the *directional trend* in hemoglobin levels. Specifically, an EPO level *higher* than expected for a given hemoglobin concentration suggests reduced erythroid activity and therefore an impending decline in hemoglobin. Conversely, an EPO level *lower* than expected reflects increased EPO clearance due to active uptake by proliferating erythroid progenitors, implying an imminent rise in hemoglobin.

This insight provides a noninvasive method for assessing bone marrow response dynamics without the need for direct sampling, such as bone marrow biopsy. Because the physiological EPO–hemoglobin relationship is approximately log-linear, representation of EPO on a logarithmic scale reflects the natural coordinate of the feedback system and may provide a more directly interpretable readout for clinical comparison across disease states. Measuring baseline EPO at the time of anemia evaluation may help contextualize hemoglobin findings, inform etiological hypotheses, and stratify patients who are more likely to benefit from exogenous EPO therapy, for example in anemia of chronic disease, where treatment response is heterogeneous. Moreover, tracking EPO levels may enable early assessment of treatment efficacy. For example, in patients with iron deficiency anemia, a decrease in EPO levels has been observed as early as three days after iron supplementation—preceding the measurable rise in hemoglobin [9]. Such early shifts in EPO may thus serve as a useful biomarker for real-time evaluation of marrow engagement or recovery. More broadly, this framework suggests that measuring EPO as part of a standardized anemia evaluation (“anemiogram”), alongside hemoglobin and conventional indices, could provide additional physiological context at diagnosis and during follow-up. These suggestions are intended as hypotheses for prospective validation rather than immediate clinical recommendations.

In this study, we focused on evaluating the model’s ability to reproduce the relationship between erythropoietin (EPO) and hemoglobin (Hb) across different disease states, as well as its dynamic response to acute blood loss. However, the model’s framework also enables prediction of EPO and Hb trajectories over time in other clinical scenarios, such as recovery following chemotherapy.

Looking ahead, the model can be extended in three meaningful directions. First, the framework could be compared to time-resolved data—such as chemotherapy-induced marrow suppression and recovery or responses to exogenous recombinant EPO—which would provide a means to further constrain model mechanisms and strengthen causal inference. Second, incorporating adaptive regulation of EPO relative to a given hemoglobin level could potentially explain phenomena such as the hypoxic–hyperoxic paradox [125]. Third, introducing a hypoxia factor to represent reduced tissue oxygenation independent of hemoglobin concentration—for example, in cardiopulmonary pathology, smoking-related hypoxemia, or altered hemoglobin–oxygen affinity —would allow the model to capture conditions in which oxygen delivery is impaired despite normal hemoglobin levels. These extensions would enable the model to generate empirically testable predictions; for instance, with a non-zero hypoxia factor, the model predicts an elevated steady-state hemoglobin level (polycythemia) and a prolonged recovery time following blood loss.

In summary, the model presented in this study demonstrates that aspects of the complex physiology of erythropoiesis in anemia states can be effectively captured using a compact set of equations. Despite its simplicity, the model reproduces key features of red blood cell regulation across both healthy and pathological states, highlighting the utility of minimal mathematical frameworks in understanding biological systems [20,21,126–128].

This study has several limitations inherent to its basis in mathematical modeling. To achieve analytical tractability and physiological interpretability, the model relies on deliberate simplifications that necessarily abstract away many biological details. Complex anemia subtypes such as hemolytic anemia, anemia of chronic disease (ACD), and chronic kidney disease (CKD) arise from multifactorial pathophysiologies involving intertwined regulatory pathways and heterogeneous patient-specific factors. Representing these processes through a small number of effective parameters may therefore obscure interactions among physiological mechanisms that contribute to disease expression and progression.

A central limitation of the present framework is its focus on steady-state behavior rather than full transient dynamics. While this allows systematic comparison across heterogeneous clinical datasets, it limits the model’s ability to capture rapid perturbations, recovery trajectories, and long-term adaptive responses. In addition, the model does not explicitly represent effects such as changes in plasma volume, which may confound concentration-based measurements of hemoglobin and circulating erythropoietin, particularly under acute conditions such as hemodilution or hemoconcentration. Moreover, because distinct physiological mechanisms may give rise to similar steady-state erythropoietin–hemoglobin relationships, agreement with steady-state data constrains the space of compatible biological explanations rather than uniquely identifying a single underlying cause. The model thus delineates classes of mechanisms consistent with observed data, rather than providing definitive causal attribution.

Additional limitations arise from the level of coarse-graining adopted. At this resolution, the model can distinguish whether erythropoietic dysfunction originates upstream or downstream of the CFU-E checkpoint, but it cannot localize defects to specific progenitor subtypes or molecular pathways within those regimes. The absence of explicit age-structured red blood cell clearance and time-dependent regulatory responses further limits the model’s ability to account for heterogeneity and delayed or history-dependent behaviors observed in iron-restricted states, inflammatory conditions, or dynamically evolving hemolytic processes.

Finally, the model lacks prospective validation. Although it was evaluated against a large body of previously published clinical data, these datasets are retrospective and heterogeneous with respect to measurement techniques, patient populations, and potential biases. Moreover, the model was calibrated to optimize agreement with available data rather than validated against independent, unseen datasets. Prospective longitudinal studies will therefore be essential to rigorously assess predictive performance and to test the model’s applicability across anemia subtypes under dynamic clinical conditions.

## Materials and methods

### Numerical simulation of the model

The model was implemented in Python version 3.9.6. At each step, the value of each variable was updated according to the Euler integration rule:


$$v_{i+\text{1}}=v_{i}+(\frac{dv}{dt})\cdot \Delta t$$

The initial conditions for H, R, C and EPO were set according to the steady state of the system.

Since all the parameters in the model are in the scale of hours, we chose a Δt of $\text{1}{\text{0}}^{\text{3}}$ seconds. The model results were the same using this time step compared to smaller values.

Each simulation ran for a default duration of $\text{2}\times \text{1}{\text{0}}^{\text{7}}$ (simulated) seconds (around 8 months) to ensure reaching steady state.

### Population simulation

To generate a more realistic, population-level behavior, we introduced stochasticity into the model by sampling the model parameters from log normal distributions. The mean of each distribution was set to the baseline parameter value listed in Table 10, while the standard deviation ranged between 5% and 30%, as specified in Table 11. The magnitude of these standard deviations was empirically chosen to reproduce the observed dispersion of erythropoietin and hemoglobin measurements in the healthy population.

**Table 10:** Model parameters, definitions, and sources.

Parameter	Description	Value	References
$\sigma_{E}[\frac{U}{s}]$	Maximal production rate of EPO	$\text{0}.\text{0}\text{3}\text{5}{\;Us}^{-\text{1}}L^{-\text{1}}$	[43–45]
D [cells]	negative inverse of the loglinear slope of EPO versus RBC, amount of C at which E production drops by 1/e	$\text{5}.\text{3}\times \text{1}{\text{0}}^{\text{1}\text{2}}\;RBC$	[43–45]
$\gamma_{E}[\frac{\text{1}}{s\cdot cells}]$	EPO degradation rate	$\text{6}.\text{1}\times \text{1}{\text{0}}^{-\text{1}\text{8}}\;s^{-\text{1}}cells^{-\text{1}}$	[15,16]
$H_{max}$	Maximum number of CFU-E cells that the bone marrow can support	$\text{1}{\text{0}}^{\text{1}\text{3}}\;cells$	[46]
$\gamma_{C}[\frac{\text{1}}{s}]$	RBC degradation rate	$\text{1}.\text{0}\cdot \text{1}{\text{0}}^{-\text{7}}s^{-\text{1}}$	[14]
$a_{max}[\frac{\text{1}}{s}]$	Maximum rate of CFU-E proliferation	$\text{5}.\text{2}\text{8}\times \text{1}{\text{0}}^{-\text{6}}s^{-\text{1}}$	[11]
$K_{a}[\frac{mU}{ml}]$	EPO concentration at which the CFU proliferation rate is half of $a_{max}$	30 mU/ml	[11]
$d_{max}[\frac{\text{1}}{s}]$	Maximum rate of CFU differentiation	$\text{1}.\text{5}\times \text{1}{\text{0}}^{-\text{6}}s^{-\text{1}}$	
$K_{d}[\frac{mU}{ml}]$	EPO concentration at which the CFU differentiation rate is half of $d_{max}$	23 mU/ml	
$\tau_{R}[s]$	Delay time it takes CFU-E to differentiate into reticulocytes	$\text{2}.\text{1}\text{6}\times \text{1}{\text{0}}^{\text{5}}\;s$	[13]
$m_{R}[\frac{\text{1}}{cells\cdot s}]$	Slope of the linear relation that converts the RBC count into the time reticulocytes remain in the marrow	$\text{1}{\text{0}}^{-\text{8}}\;(cells\cdot s{)}^{-\text{1}}$	[14]
$n_{R}[\frac{\text{1}}{s}]$	Baseline (intercept) time that reticulocytes stay in the bone marrow	$\text{5}.\text{8}\times \text{1}{\text{0}}^{\text{4}}\;s^{-\text{1}}$	[14]

**Table 11:** Standard deviations assigned to model parameters for population-level simulations.

parameter	$\gamma_{C}$	$\gamma_{E}$	$H_{max}$	$\sigma_{E}$	D	$a_{max}{,d}_{max}$	$k_{a}{,k}_{d}$
Standard deviation	0.2	0.3	0.3	0.1	0.05	0.1	0.2

### Data collection

In most cases, original data were not provided in tabular form but embedded in figures. To extract numerical values, we used a web-based digitizing tool [129] to convert graphical data into analyzable format and manually checked. All values were standardized to hemoglobin concentration in g/dL. When data were reported as hematocrit (Hct), values were converted to hemoglobin using the relation Hct = 3 × Hb.

### Quantification of the fit between experimental data points and simulation data points

The Wasserstein distance [51], also known as the Earth Mover’s Distance, quantifies the dissimilarity between two probability distributions by measuring the minimal cost required to transform one distribution into the other. To normalize the distance to a meaningful unit of standard deviation, both experimental and simulated data were scaled by the standard deviation of the experimental data, in each axis separately. As a reference, we used the distance between the model and experimental data from the healthy population. To minimize the impact of outliers, we excluded bioassay measurements that were frequently aberrant (see Figs 4–9).

## Supporting information

S1 File Estimation of model parameter values. This file provides detailed derivations and literature-based estimates for all model parameters, except d_max_ and K_d_, and discusses the indirect and uncertain nature of available measurements for H_max_.
(DOCX)

S2 File Estimation of the circulating reticulocyte percentage from model variables. This file describes how the circulating reticulocyte percentage can be inferred from the model’s marrow reticulocyte pool R(t) by decomposing the RBC compartment into circulating reticulocytes and mature erythrocytes, using clinically derived transition rates based on the reticulocyte maturation correction framework.
(DOCX)

## References

[1] Billett HH. Hemoglobin and Hematocrit. In: Walker HK, Hall WD, Hurst JW, editors. Clinical Methods: The History, Physical, and Laboratory Examinations [Internet]. 3rd ed. Boston: Butterworths; 1990 [cited 2025 Oct 15]. Available from: http://www.ncbi.nlm.nih.gov/books/NBK259/ 21250045

[2] Adamson JW, Longo DL. Anemia and Polycythemia. In: Jameson JL, Fauci AS, Kasper DL, Hauser SL, Longo DL, Loscalzo J, editors. Harrison’s Principles of Internal Medicine [Internet]. 20th ed. New York, NY: McGraw-Hill Education; 2018 [cited 2025 Oct 15]. Available from: accessmedicine.mhmedical.com/content.aspx?aid=1160011376

[3] McLeod DL, Shreeve MM, Axelrad AA. Improved plasma culture system for production of erythrocytic colonies in vitro: quantitative assay method for CFU-E. Blood. 1974;44(4):517–34. 4137721

[4] Koury MJ, Bondurant MC. Erythropoietin retards DNA breakdown and prevents programmed death in erythroid progenitor cells. Science. 1990;248(4953):378–81. doi: 10.1126/science.2326648 2326648

[5] Koury ST, Bondurant MC, Koury MJ. Localization of erythropoietin synthesizing cells in murine kidneys by in situ hybridization. Blood. 1988;71(2):524–7. doi: 10.1182/blood.v71.2.524.524 3337914

[6] Lacombe C, Da Silva JL, Bruneval P, Fournier JG, Wendling F, Casadevall N, et al. Peritubular cells are the site of erythropoietin synthesis in the murine hypoxic kidney. J Clin Invest. 1988;81(2):620–3. doi: 10.1172/JCI113363 3339134 PMC329613

[7] Maxwell PH, Osmond MK, Pugh CW, Heryet A, Nicholls LG, Tan CC, et al. Identification of the renal erythropoietin-producing cells using transgenic mice. Kidney Int. 1993;44(5):1149–62. doi: 10.1038/ki.1993.362 8264149

[8] Bora N, Elliott SG, Foote M, Molineux G. Erythropoietins, erythropoietic factors, and erythropoiesis. Molecular, cellular, preclinical, and clinical biology: 2nd revised and extended edition, 2009, 348 p., Hardcover, Birkhäuser Verlag AG, Basel, Switzerland, ISBN 978-3-7643-8694-8. Inflamm Res. 2010;59(6):489–90. doi: 10.1007/s00011-010-0186-4

[9] Cazzola M, Guarnone R, Cerani P, Centenara E, Rovati A, Beguin Y. Red blood cell precursor mass as an independent determinant of serum erythropoietin level. Blood. 1998;91(6):2139–45. doi: 10.1182/blood.v91.6.2139 9490701

[10] Bunn HF. Erythropoietin. Cold Spring Harb Perspect Med. 2013;3(3):a011619. 10.1101/cshperspect.a011619 PMC3579209 23457296

[11] Sawada K, Krantz SB, Kans JS, Dessypris EN, Sawyer S, Glick AD, et al. Purification of human erythroid colony-forming units and demonstration of specific binding of erythropoietin. J Clin Invest. 1987;80(2):357–66. doi: 10.1172/JCI113080 3038955 PMC442245

[12] Broudy VC, Lin N, Brice M, Nakamoto B, Papayannopoulou T. Erythropoietin receptor characteristics on primary human erythroid cells. Blood. 1991;77(12):2583–90. doi: 10.1182/blood.v77.12.2583.bloodjournal77122583 1646044

[13] Hattangadi SM, Wong P, Zhang L, Flygare J, Lodish HF. From stem cell to red cell: regulation of erythropoiesis at multiple levels by multiple proteins, RNAs, and chromatin modifications. Blood. 2011;118(24):6258–68. doi: 10.1182/blood-2011-07-356006 21998215 PMC3236116

[14] Prchal JT, Thiagarajan P. Erythropoiesis and Red Cell Turnover. In: Kaushansky K, Prchal JT, Burns LJ, Lichtman MA, Levi M, Linch DC, editors. Williams Hematology, 10e [Internet]. New York, NY: McGraw-Hill Education; 2021. Available from: hemonc.mhmedical.com/content.aspx?aid=1178739323

[15] Jelkmann W. Erythropoietin: structure, control of production, and function. Physiol Rev. 1992;72(2):449–89. doi: 10.1152/physrev.1992.72.2.449 1557429

[16] Bélair J, Mackey MC, Mahaffy JM. Age-structured and two-delay models for erythropoiesis. Math Biosci. 1995;128(1–2):317–46. doi: 10.1016/0025-5564(94)00078-e 7606142

[17] Gardner WM, Razo C, McHugh TA, Hagins H, Vilchis-Tella VM, Hennessy C. Prevalence, years lived with disability, and trends in anaemia burden by severity and cause, 1990–2021: findings from the Global Burden of Disease Study 2021. Lancet Haematol. 2023;10(9):e713-34. doi: 10.1016/S2352-3026(23)00160-6 37536353 PMC10465717

[18] Pasqualetti P, Collacciani A, Casale R. Circadian rhythm of serum erythropoietin in myelodysplastic syndromes. Eur Rev Med Pharmacol Sci. 2000;4(5–6):111–5. 11710507

[19] Dietrich JW, Landgrafe-Mende G, Wiora E, Chatzitomaris A, Klein HH, Midgley JEM, et al. Calculated Parameters of Thyroid Homeostasis: Emerging Tools for Differential Diagnosis and Clinical Research. Front Endocrinol (Lausanne). 2016;7:57. doi: 10.3389/fendo.2016.00057 27375554 PMC4899439

[20] Korem Kohanim Y, Milo T, Raz M, Karin O, Bar A, Mayo A, et al. Dynamics of thyroid diseases and thyroid-axis gland masses. Mol Syst Biol. 2022;18(8):e10919. doi: 10.15252/msb.202210919 35938225 PMC9358402

[21] Alon U. Systems Medicine: Physiological Circuits and the Dynamics of Disease. New York: Chapman and Hall/CRC; 2023. p. 270. doi: 10.1201/9781003356929

[22] Keener JP. Mathematical physiology. 2nd ed. New York: Springer; 2009.

[23] Higgins JM, Mahadevan L. Physiological and pathological population dynamics of circulating human red blood cells. Proc Natl Acad Sci U S A. 2010;107(47):20587–92. doi: 10.1073/pnas.1012747107 21059904 PMC2996693

[24] Foy BH, Sundt TM, Carlson JCT, Aguirre AD, Higgins JM. Human acute inflammatory recovery is defined by co-regulatory dynamics of white blood cell and platelet populations. Nat Commun. 2022;13(1):4705. doi: 10.1038/s41467-022-32222-2 35995789 PMC9395541

[25] Chaudhury A, Miller GD, Eichner D, Higgins JM. Single-cell modeling of routine clinical blood tests reveals transient dynamics of human response to blood loss. Barkai N, Obermeyer Z, Spitalnik S, editors. eLife. 2019;8:e48590. doi: 10.7554/eLife.48590 PMC6917488 31845889

[26] Savill NJ, Chadwick W, Reece SE. Quantitative analysis of mechanisms that govern red blood cell age structure and dynamics during anaemia. PLoS Comput Biol. 2009;5(6):e1000416. doi: 10.1371/journal.pcbi.1000416 PMC2694369 19557192

[27] Bouchnita A, Rocca A, Fanchon E, Koury MJ, Moulis JM, Volpert V. Multi-scale modelling of erythropoiesis and hemoglobin production. J Inorg Organomet Polym Mater. 2016;26(6):1362–79. doi: 10.1007/s10904-016-0437-0

[28] Fuertinger DH, Kappel F, Thijssen S, Levin NW, Kotanko P. A model of erythropoiesis in adults with sufficient iron availability. J Math Biol. 2013;66(6):1209–40. doi: 10.1007/s00285-012-0530-0 22526838

[29] Loeffler M, Pantel K, Wulff H, Wichmann HE. A mathematical model of erythropoiesis in mice and rats. Part 1: Structure of the model. Cell Tissue Kinet. 1989;22(1):13–30. doi: 10.1111/j.1365-2184.1989.tb00198.x 2790923

[30] Crauste F, Pujo-Menjouet L, Génieys S, Molina C, Gandrillon O. Adding self-renewal in committed erythroid progenitors improves the biological relevance of a mathematical model of erythropoiesis. J Theor Biol. 2008;250(2):322–38. doi: 10.1016/j.jtbi.2007.09.041 17997418

[31] Tetschke M, Lilienthal P, Pottgiesser T, Fischer T, Schalk E, Sager S. Mathematical Modeling of RBC Count Dynamics after Blood Loss. Processes. 2018;6(9):157. doi: 10.3390/pr6090157

[32] Jansson LT, Perkkio MV, Clemons G, Refino CJ, Dallman PR. Erythropoietin concentration during the development and recovery from iron deficiency in the rat. Blood. 1985;65(4):959. 3978235

[33] Birgegård G, Wide L, Simonsson B. Marked erythropoietin increase before fall in Hb after treatment with cytostatic drugs suggests mechanism other than anaemia for stimulation. Br J Haematol. 1989;72(3):462–6. doi: 10.1111/j.1365-2141.1989.tb07733.x 2669930

[34] Cazzola M, Mercuriali F, Brugnara C. Use of recombinant human erythropoietin outside the setting of uremia. Blood. 1997;89(12):4248–67. doi: 10.1182/blood.v89.12.4248 9192747

[35] Beguin Y, Clemons GK, Pootrakul P, Fillet G. Quantitative assessment of erythropoiesis and functional classification of anemia based on measurements of serum transferrin receptor and erythropoietin [Internet]. 1993 [cited 2025 Jun 29]. Available from: https://ashpublications.org/blood/article-abstract/81/4/1067/169782 8427988

[36] Barosi G. Inadequate erythropoietin response to anemia: definition and clinical relevance. Ann Hematol. 1994;68(5):215–23. doi: 10.1007/BF01737420 8018762

[37] Ludwig H, Sundal E, Pecherstorfer M, Leitgeb C, Bauernhofer T, Beinhauer A, et al. Recombinant human erythropoietin for the correction of cancer associated anemia with and without concomitant cytotoxic chemotherapy. Cancer. 1995;76(11):2319–29. doi: 10.1002/1097-0142(19951201)76:11<2319::aid-cncr2820761121>3.0.co;2-u 8635038

[38] Ludwig H, Fritz E, Leitgeb C, Pecherstorfer M, Samonigg H, Schuster J. Prediction of response to erythropoietin treatment in chronic anemia of cancer. Blood. 1994;84(4):1056–63. 7741835

[39] Schapira L, Antin JH, Ransil BJ, Antman KH, Eder JP, McGarigle CJ. Serum erythropoietin levels in patients receiving intensive chemotherapy and radiotherapy. Blood. 1990;76(11):2354–9. 2257306

[40] Shemin D, Rittenberg D. The life span of the human red blood cell. J Biol Chem. 1946;166(2):627–36. doi: 10.1016/s0021-9258(17)35201-8 20276177

[41] Shrestha RP, Horowitz J, Hollot CV, Germain MJ, Widness JA, Mock DM, et al. Models for the red blood cell lifespan. J Pharmacokinet Pharmacodyn. 2016;43(3):259–74. doi: 10.1007/s10928-016-9470-4 27039311 PMC4887310

[42] Dohnhorst AC. The interpretation of red cell survival curves. Blood. 1951;6(12):1284–92. doi: 10.1182/blood.V6.12.1284.1284 14886401

[43] Erslev AJ, Caro J, Miller O, Silver R. Plasma erythropoietin in health and disease. Ann Clin Lab Sci. 1980;10(3):250–7. 7396390

[44] Artunc F, Risler T. Serum erythropoietin concentrations and responses to anaemia in patients with or without chronic kidney disease. Nephrol Dial Transplant. 2007;22(10):2900–8. doi: 10.1093/ndt/gfm316 17556407

[45] Bergamaschi G, Markopoulos K, Albertini R, Di Sabatino A, Biagi F, Ciccocioppo R, et al. Anemia of chronic disease and defective erythropoietin production in patients with celiac disease. Haematologica. 2008;93(12):1785–91. doi: 10.3324/haematol.13255 18815191

[46] Iscove NN. The role of erythropoietin in regulation of population size and cell cycling of early and late erythroid precursors in mouse bone marrow. Cell Tissue Kinet. 1977;10(4):323–34. doi: 10.1111/j.1365-2184.1977.tb00300.x 884703

[47] Sherwood JB, Goldwasser E, Chilcote R, Carmichael LD, Nagel RL. Sickle cell anemia patients have low erythropoietin levels for their degree of anemia. Blood. 1986;67(1):46–9. doi: 10.1182/blood.v67.1.46.bloodjournal67146 3940552

[48] Fattizzo B, Michel M, Zaninoni A, Giannotta J, Guillet S, Frederiksen H, et al. Efficacy of recombinant erythropoietin in autoimmune hemolytic anemia: a multicenter international study. Haematologica. 2021;106(2):622–5. doi: 10.3324/haematol.2020.250522 32354865 PMC7849557

[49] Morgan AG, Gruber CA, Serjeant GR. Erythropoietin and renal function in sickle-cell disease. Br Med J (Clin Res Ed). 1982;285(6356):1686–8. doi: 10.1136/bmj.285.6356.1686 6816331 PMC1500701

[50] Camaschella C, Gonella S, Calabrese R, Vischia F, Roetto A, Graziadei G, et al. Serum erythropoietin and circulating transferrin receptor in thalassemia intermedia patients with heterogeneous genotypes. Haematologica. 1996;81(5):397–403. 8952151

[51] Rüschendorf L. The Wasserstein distance and approximation theorems. Z Wahrscheinlichkeitstheorie verw Gebiete. 1985;70(1):117–29. doi: 10.1007/bf00532240

[52] Kiss JE, Brambilla D, Glynn SA, Mast AE, Spencer BR, Stone M, et al. Oral iron supplementation after blood donation: a randomized clinical trial. JAMA. 2015;313(6):575–83. doi: 10.1001/jama.2015.119 25668261 PMC5094173

[53] Meulenbeld A, Turkulainen EV, Li W, Pothast MR, Qi H, Allara E, et al. Blood donor populations reveal a clear association between ferritin and change in haemoglobin levels. Br J Haematol. 2025;207(3):1096–103. doi: 10.1111/bjh.70066 40770905 PMC12436240

[54] Schrezenmeier H, Noé G, Raghavachar A, Rich IN, Heimpel H, Kubanek B. Serum erythropoietin and serum transferrin receptor levels in aplastic anaemia. Br J Haematol. 1994;88(2):286–94. doi: 10.1111/j.1365-2141.1994.tb05020.x 7803272

[55] Schreiber S, Howaldt S, Schnoor M, Nikolaus S, Bauditz J, Gasché C, et al. Recombinant erythropoietin for the treatment of anemia in inflammatory bowel disease. N Engl J Med. 1996;334(10):619–23. doi: 10.1056/NEJM199603073341002 8592524

[56] Wallner SF, Kurnick JE, Vautrin RM, White MJ, Chapman RG, Ward HP. Levels of erythropoietin in patients with the anemias of chronic diseases and liver failure. Am J Hematol. 1977;3:37–44. doi: 10.1002/ajh.2830030105 602942

[57] Das RE, Milne A, Rowley M, Smith EC, Cotes PM. Serum immunoreactive erythropoietin in patients with idiopathic aplastic and Fanconi’s anaemias. Br J Haematol. 1992;82(3):601–7. doi: 10.1111/j.1365-2141.1992.tb06474.x 1486041

[58] Erslev AJ, Wilson J, Caro J. Erythropoietin titers in anemic, nonuremic patients. J Lab Clin Med. 1987;109(4):429–33. 3102659

[59] Jelkmann W, Wiedemann G. Serum erythropoietin level: relationships to blood hemoglobin concentration and erythrocytic activity of the bone marrow. Klin Wochenschr. 1990;68(8):403–7. doi: 10.1007/BF01648581 2348644

[60] Fandrey J, Jelkmann WE. Interleukin-1 and tumor necrosis factor-alpha inhibit erythropoietin production in vitro. Ann N Y Acad Sci. 1991;628:250–5. doi: 10.1111/j.1749-6632.1991.tb17252.x 1712553

[61] Gascon P, Scala G. Decreased interleukin-1 production in aplastic anemia. Am J Med. 1988;85(5):668–74. doi: 10.1016/S0002-9343(88)80240-7 3263800

[62] Aoki I, Higashi K, Homori M, Chikazawa H, Ishikawa K. Responsiveness of bone marrow erythropoietic stem cells (CFU-E and BFU-E) to recombinant human erythropoietin (rh-Ep) in vitro in aplastic anemia and myelodysplastic syndrome. Am J Hematol. 1990;35(1):6–12. doi: 10.1002/ajh.2830350103 2389770

[63] Piroso E, Erslev AJ, Flaharty KK, Caro J. Erythropoietin life span in rats with hypoplastic and hyperplastic bone marrows. Am J Hematol. 1991;36(2):105–10. doi: 10.1002/ajh.2830360208 2012061

[64] Lezon C, Alippi RM, Barcelo AC, Martinez MP, Conti MI, Bozzini CE. Depression of stimulated erythropoietin production in mice with enhanced erythropoiesis. Haematologica. 1995;80(6):491–4. doi: 10.3324/%x 8647512

[65] Alexanian R. Erythropoietin excretion in bone marrow failure and hemolytic anemia. J Lab Clin Med. 1973;82(3):438–45. 4728292

[66] Panjeta M, Tahirović I, Sofić E, Ćorić J, Dervišević A. Interpretation of Erythropoietin and Haemoglobin Levels in Patients with Various Stages of Chronic Kidney Disease. J Med Biochem. 2017;36(2):145–52. doi: 10.1515/jomb-2017-0014 28680358 PMC5471647

[67] Kubo K, Okamura T, Sugiyama D, Hisamatsu T, Hirata A, Kadota A, et al. Effect of Chronic Kidney Disease or Anemia or Both on Cardiovascular Mortality in a 25-Year Follow-Up Study of Japanese General Population (From NIPPON DATA90). Am J Cardiol. 2022;184:1–6. doi: 10.1016/j.amjcard.2022.08.027 36127178

[68] McGonigle RJ, Wallin JD, Shadduck RK, Fisher JW. Erythropoietin deficiency and inhibition of erythropoiesis in renal insufficiency. Kidney Int. 1984;25(2):437–44. doi: 10.1038/ki.1984.36 6727139

[69] Gutman RA, Huang AT. Inhibitor of marrow thymidine incorporation from sera of patients with uremia. Kidney Int. 1980;18(6):715–24. doi: 10.1038/ki.1980.190 7206456

[70] Shaw AB. Haemolysis in chronic renal failure. Br Med J. 1967;2(5546):213–6. doi: 10.1136/bmj.2.5546.213 6023106 PMC1841174

[71] McMurray J, Parfrey P, Adamson JW, Aljama P, Berns JS, Bohlius J, et al. Kidney disease: Improving global outcomes (KDIGO) anemia work group. KDIGO clinical practice guideline for anemia in chronic kidney disease [Internet]. 2012. 10.1038/kisup.2012.37

[72] Fehr T, Ammann P, Garzoni D, Korte W, Fierz W, Rickli H, et al. Interpretation of erythropoietin levels in patients with various degrees of renal insufficiency and anemia. Kidney Int. 2004;66(3):1206–11. doi: 10.1111/j.1523-1755.2004.00880.x 15327419

[73] Ginouvès A, Ilc K, Macías N, Pouysségur J, Berra E. PHDs overactivation during chronic hypoxia “desensitizes” HIFα and protects cells from necrosis. Proceedings of the National Academy of Sciences. 2008;105(12):4745–50. doi: 10.1073/pnas.0705680105 PMC2290777 18347341

[74] Vaziri ND, Kaupke CJ, Barton CH, Gonzales E. Plasma concentration and urinary excretion of erythropoietin in adult nephrotic syndrome. Am J Med. 1992;92(1):35–40. doi: 10.1016/0002-9343(92)90012-z 1731507

[75] Widness JA, Veng-Pedersen P, Schmidt RL, Lowe LS, Kisthard JA, Peters C. In vivo 125I-erythropoietin pharmacokinetics are unchanged after anesthesia, nephrectomy and hepatectomy in sheep. J Pharmacol Exp Ther. 1996;279(3):1205–10. doi: 10.1016/s0022-3565(25)21278-2 8968342

[76] Coles GA, Liberek T, Davies ME, Robinson M, Jones J, Thomas G, et al. Estimation of erythropoietin secretion rate in normal and uremic subjects. Am J Physiol. 1992;263(5 Pt 2):F939-44. doi: 10.1152/ajprenal.1992.263.5.F939 1443181

[77] Thevenod F, Radtke HW, Grützmacher P, Vincent E, Koch KM, Schoeppe W, et al. Deficient feedback regulation of erythropoiesis in kidney transplant patients with polycythemia. Kidney Int. 1983;24(2):227–32. doi: 10.1038/ki.1983.148 6355616

[78] Mason-Garcia M, Beckman BS, Brookins JW, Powell JS, Lanham W, Blaisdell S, et al. Development of a new radioimmunoassay for erythropoietin using recombinant erythropoietin. Kidney Int. 1990;38(5):969–75. doi: 10.1038/ki.1990.299 2266682

[79] Gowanlock Z, Sriram S, Martin A, Xenocostas A, Lazo-Langner A. Erythropoietin Levels in Elderly Patients with Anemia of Unknown Etiology. PLoS One. 2016;11(6):e0157279. doi: 10.1371/journal.pone.0157279 27310832 PMC4911007

[80] Caro J, Brown S, Miller O, Murray T, Erslev AJ. Erythropoietin levels in uremic nephric and anephric patients. J Lab Clin Med. 1979;93(3):449–58. 570997

[81] Radtke HW, Claussner A, Erbes PM, Scheuermann EH, Schoeppe W, Koch KM. Serum erythropoietin concentration in chronic renal failure: relationship to degree of anemia and excretory renal function. Blood. 1979;54(4):877–84. doi: 10.1182/blood.v54.4.877.bloodjournal544877 476305

[82] Hsieh C-C, Chan M-J, Su Y-J, Fu J-F, Wang I-K, Chen C-Y, et al. Bone Marrow Hypocellularity in Patients with End-Stage Kidney Disease. Healthcare (Basel). 2021;9(11):1452. doi: 10.3390/healthcare9111452 34828498 PMC8621268

[83] Singh S, Bhatta S. Biochemical and hematological parameters in chronic kidney disease. J Manmohan Meml Inst Health Sci. 2018;4(1):4–11. doi: 10.3126/jmmihs.v4i1.21132

[84] Theurl I, Mattle V, Seifert M, Mariani M, Marth C, Weiss G. Dysregulated monocyte iron homeostasis and erythropoietin formation in patients with anemia of chronic disease. Blood. 2006;107(10):4142–8. doi: 10.1182/blood-2005-08-3364 16434484

[85] Hutcheon AW, Horton PW, Orr JS, Dagg JH. The assessment of red cell survival in normal subjects and in patients with haemolytic disorders and ineffective erythropoiesis using the radioiron occupancy method. Br J Haematol. 1977;37(2):195–205. doi: 10.1111/j.1365-2141.1977.tb06835.x 603754

[86] McCurdy P, Mahmood L, Sherman A. Red cell life span in sickle cell-hemoglobin C disease with a note about sickle cell-hemoglobin O Arab. Blood. 1975;45(2):273–9. doi: 10.1182/blood.V45.2.273.273 1120186

[87] Baer AN, Dessypris EN, Krantz SB. The pathogenesis of anemia in rheumatoid arthritis: a clinical and laboratory analysis. Semin Arthritis Rheum. 1990;19(4):209–23. doi: 10.1016/0049-0172(90)90001-v 2181669

[88] Dinant HJ, de Maat CE. Erythropoiesis and mean red-cell lifespan in normal subjects and in patients with the anaemia of active rheumatoid arthritis. Br J Haematol. 1978;39(3):437–44. doi: 10.1111/j.1365-2141.1978.tb01114.x 698120

[89] Ludwiczek S, Aigner E, Theurl I, Weiss G. Cytokine-mediated regulation of iron transport in human monocytic cells. Blood. 2003;101(10):4148–54. doi: 10.1182/blood-2002-08-2459 12522003

[90] Nemeth E, Tuttle MS, Powelson J, Vaughn MB, Donovan A, Ward DM. Hepcidin regulates cellular iron efflux by binding to ferroportin and inducing its internalization. Science. 2004;306(5704):2090–3. doi: 10.1126/science.1104742 15514116

[91] Ganz T, Nemeth E. Hepcidin and iron homeostasis. Biochim Biophys Acta. 2012;1823(9):1434–43. doi: 10.1016/j.bbamcr.2012.01.014 22306005 PMC4048856

[92] Means RJ, Krantz S. Progress in understanding the pathogenesis of the anemia of chronic disease. Blood. 1992;80(7):1639–47. doi: 10.1182/blood.V80.7.1639.1639 1391934

[93] Bárány P. Inflammation, serum C-reactive protein, and erythropoietin resistance. Nephrol Dial Transplant. 2001;16(2):224–7. doi: 10.1093/ndt/16.2.224 11158392

[94] Taniguchi S, Dai CH, Price JO, Krantz SB. Interferon gamma downregulates stem cell factor and erythropoietin receptors but not insulin-like growth factor-I receptors in human erythroid colony-forming cells. Blood. 1997;90(6):2244–52. 9310475

[95] Schooley JC, Kullgren B, Allison AC. Inhibition by interleukin-1 of the action of erythropoietin on erythroid precursors and its possible role in the pathogenesis of hypoplastic anaemias. Br J Haematol. 1987;67(1):11–7. doi: 10.1111/j.1365-2141.1987.tb02289.x 3499170

[96] Dainiak N, Kulkarni V, Howard D, Kalmanti M, Dewey MC, Hoffman R. Mechanisms of abnormal erythropoiesis in malignancy. Cancer. 1983;51(6):1101–6. doi: 10.1002/1097-0142(19830315)51:6<1101::aid-cncr2820510622>3.0.co;2-g 6821869

[97] Miller CB, Jones RJ, Piantadosi S, Abeloff MD, Spivak JL. Decreased erythropoietin response in patients with the anemia of cancer. N Engl J Med. 1990;322(24):1689–92. doi: 10.1056/NEJM199006143222401 2342534

[98] Spivak JL. Cancer-related anemia: its causes and characteristics. Semin Oncol. 1994;21(2 Suppl 3):3–8. 8202724

[99] Opasich C, Cazzola M, Scelsi L, De Feo S, Bosimini E, Lagioia R, et al. Blunted erythropoietin production and defective iron supply for erythropoiesis as major causes of anaemia in patients with chronic heart failure. Eur Heart J. 2005;26(21):2232–7. doi: 10.1093/eurheartj/ehi388 15987710

[100] Fischl M, Galpin JE, Levine JD, Groopman JE, Henry DH, Kennedy P, et al. Recombinant human erythropoietin for patients with AIDS treated with zidovudine. N Engl J Med. 1990;322(21):1488–93. doi: 10.1056/NEJM199005243222103 2186273

[101] Faquin WC, Schneider TJ, Goldberg MA. Effect of inflammatory cytokines on hypoxia-induced erythropoietin production. Blood. 1992;79(8):1987–94. doi: 10.1182/blood.v79.8.1987.1987 1373333

[102] Vannucchi AM, Grossi A, Rafanelli D, Statello M, Cinotti S, Rossi-Ferrini P. Inhibition of erythropoietin production in vitro by human interferon gamma. Br J Haematol. 1994;87(1):18–23. doi: 10.1111/j.1365-2141.1994.tb04864.x 7947242

[103] Caligaris-Cappio F, Bergui L, Tesio L, Ziano R, Camussi G. HLA-Dr+ T cells of the Leu 3 (helper) type infiltrate the kidneys of patients with systemic lupus erythematosus. Clin Exp Immunol. 1985;59(1):185–9. 3156013 PMC1577155

[104] Souma T, Yamazaki S, Moriguchi T, Suzuki N, Hirano I, Pan X, et al. Plasticity of renal erythropoietin-producing cells governs fibrosis. J Am Soc Nephrol. 2013;24(10):1599–616. doi: 10.1681/ASN.2013010030 23833259 PMC3785278

[105] Pinto JP, Ribeiro S, Pontes H, Thowfeequ S, Tosh D, Carvalho F. Erythropoietin mediates hepcidin expression in hepatocytes through EPOR signaling and regulation of C/EBPalpha. Blood. 2008;111(12):5727–33. doi: 10.1182/blood-2007-08-106195 18326822 PMC2597200

[106] Schett G, Firbas U, Füreder W, Hiesberger H, Winkler S, Wachauer D, et al. Decreased serum erythropoietin and its relation to anti-erythropoietin antibodies in anaemia of systemic lupus erythematosus. Rheumatology (Oxford). 2001;40(4):424–31. doi: 10.1093/rheumatology/40.4.424 11312382

[107] Ferrucci L, Guralnik JM, Woodman RC, Bandinelli S, Lauretani F, Corsi AM, et al. Proinflammatory state and circulating erythropoietin in persons with and without anemia. Am J Med. 2005;118(11):1288. doi: 10.1016/j.amjmed.2005.06.039 16271918

[108] Macdougall IC, Cooper AC. Erythropoietin resistance: the role of inflammation and pro-inflammatory cytokines. Nephrol Dial Transplant. 2002;17 Suppl 11:39–43. doi: 10.1093/ndt/17.suppl_11.39 12386257

[109] Pincus T, Olsen NJ, Russell IJ, Wolfe F, Harris ER, Schnitzer TJ, et al. Multicenter study of recombinant human erythropoietin in correction of anemia in rheumatoid arthritis. Am J Med. 1990;89(2):161–8. doi: 10.1016/0002-9343(90)90294-n 2200263

[110] Peeters HR, Jongen-Lavrencic M, Vreugdenhil G, Swaak AJ. Effect of recombinant human erythropoietin on anaemia and disease activity in patients with rheumatoid arthritis and anaemia of chronic disease: a randomised placebo controlled double blind 52 weeks clinical trial. Ann Rheum Dis. 1996;55(10):739–44. doi: 10.1136/ard.55.10.739 8984939 PMC1010292

[111] Henry DH, Beall GN, Benson CA, Carey J, Cone LA, Eron LJ, et al. Recombinant human erythropoietin in the treatment of anemia associated with human immunodeficiency virus (HIV) infection and zidovudine therapy. Overview of four clinical trials. Ann Intern Med. 1992;117(9):739–48. doi: 10.7326/0003-4819-117-9-739 1416576

[112] Abels R, Larholt K, Krantz K, Bryant E. Recombinant Human Erythropoietin (rHuEPO) for the Treatment of the Anemia of Cancer. Oncologist. 1996;1(3):140–50. doi: 10.1634/theoncologist.1-3-140 10387980

[113] Camacho J, Arnalich F, Zamorano AF, Vázquez JJ. Serum erythropoietin levels in the anaemia of chronic disorders. J Intern Med. 1991;229(1):49–54. doi: 10.1111/j.1365-2796.1991.tb00305.x 1995763

[114] Cox R, Musial T, Gyde OH. Reduced erythropoietin levels as a cause of anaemia in patients with lung cancer. Eur J Cancer Clin Oncol. 1986;22(4):511–4. doi: 10.1016/0277-5379(86)90120-3 3732355

[115] Hochberg MC, Arnold CM, Hogans BB, Spivak JL. Serum immunoreactive erythropoietin in rheumatoid arthritis: impaired response to anemia. Arthritis Rheum. 1988;31(10):1318–21. doi: 10.1002/art.1780311016 3178910

[116] Kendall R, Wasti A, Harvey A, Hill J, Chapman C, Norfolk DR. The relationship of haemoglobin to serum erythropoietin concentrations in the anaemia of rheumatoid arthritis: the effect of oral prednisolone. Rheumatology. 1993;32(3):204–8. doi: 10.1093/rheumatology/32.3.204 8448609

[117] Nielsen OJ, Andersen LS, Ludwigsen E, Bouchelouche P, Hansen TM, Birgens H, et al. Anaemia of rheumatoid arthritis: serum erythropoietin concentrations and red cell distribution width in relation to iron status. Ann Rheum Dis. 1990;49(6):349–53. doi: 10.1136/ard.49.6.349 2383057 PMC1004098

[118] Spivak JL, Barnes DC, Fuchs E, Quinn TC. Serum immunoreactive erythropoietin in HIV-infected patients. JAMA. 1989;261(21):3104–7. 2716142

[119] Vreugdenhil G, Wognum AW, van Eijk HG, Swaak AJ. Anaemia in rheumatoid arthritis: the role of iron, vitamin B12, and folic acid deficiency, and erythropoietin responsiveness. Ann Rheum Dis. 1990;49(2):93–8. doi: 10.1136/ard.49.2.93 2317122 PMC1003985

[120] Wood PA, Hrushesky WJ. Cisplatin-associated anemia: an erythropoietin deficiency syndrome. J Clin Invest. 1995;95(4):1650–9. doi: 10.1172/JCI117840 7706473 PMC295669

[121] Keel SB, Doty R, Liu L, Nemeth E, Cherian S, Ganz T, et al. Evidence that the expression of transferrin receptor 1 on erythroid marrow cells mediates hepcidin suppression in the liver. Exp Hematol. 2015;43(6):469-78.e6. doi: 10.1016/j.exphem.2015.03.001 25782630 PMC4771411

[122] Estrov Z, Cohen A, Gelfand EW, Freedman MH. In vitro cytotoxicity of deferoxamine on human marrow haematopoietic progenitors. Toxicol In Vitro. 1988;2(2):131–4. doi: 10.1016/0887-2333(88)90024-0 20702348

[123] Nocka KH, Pelus LM. Cell cycle specific effects of deferoxamine on human and murine hematopoietic progenitor cells. Cancer Res. 1988;48(13):3571–5. 3378203

[124] Orphanidou-Vlachou E, Tziakouri-Shiakalli C, Georgiades CS. Extramedullary hemopoiesis. Semin Ultrasound CT MRI. 2014;35(3):255–62. doi: 10.1053/j.sult.2013.12.001 24929265

[125] Hadanny A, Efrati S. The hyperoxic-hypoxic paradox. Biomolecules. 2020;10(6):958. doi: 10.3390/biom10060958 32630465 PMC7355982

[126] Alon U. An Introduction to Systems Biology: Design Principles of Biological Circuits. 2nd ed. New York: Chapman and Hall/CRC; 2019. p. 342. doi: 10.1201/9780429283321

[127] Topp B, Promislow K, deVries G, Miura RM, Finegood DT. A model of beta-cell mass, insulin, and glucose kinetics: pathways to diabetes. J Theor Biol. 2000;206(4):605–19. doi: 10.1006/jtbi.2000.2150 11013117

[128] Vinther F, Andersen M, Ottesen JT. The minimal model of the hypothalamic–pituitary–adrenal axis. J Math Biol. 2011;63(4):663–90. doi: 10.1007/s00285-010-0384-2 21107577

[129] PlotDigitizer Online App [Internet]. [cited 2026 Mar 6]. Available from: https://plotdigitizer.com/app

