# Supplementary material for: A minimal mathematical model of red blood cell homeostasis in anemia
Source: PLoS Comput Biol. 2026 Mar 24;22(3):e1014111. doi: 10.1371/journal.pcbi.1014111 (PMC13048500; doi:10.1371/journal.pcbi.1014111)
Supplement: S1 File — This file provides detailed derivations and literature-based estimates for all model parameters, except dmax and Kd, and discusses the indirect and uncertain nature of available measurements for Hmax. (DOCX) [file pcbi.1014111.s001.docx]

**A minimal mathematical model of red blood cell homeostasis in anemia**

**Supporting information – S1 File**

Herut Dor^1,*^*,* Uri Alon^2,**^

^1^Gray Faculty of Medical and Health Sciences, Tel Aviv University, Tel Aviv, Israel

^2^Department of Molecular Cell Biology, Weizmann Institute of Science, Rehovot, Israel

*Corresponding author

E-mail:[herutdor@gmail.com](mailto:herutdor@gmail.com) (HD)

**Corresponding author

E-mail:[uri.alon@weizmann.ac.il](mailto:uri.alon@weizmann.ac.il) (UA)

**S1 File: Estimation of model parameter values**

The red blood cell removal rate $\gamma_{C}$ was estimated as follows, based on (1). Under uniform distribution of RBC age, $\gamma_{C}=\frac{1}{120 days}=\frac{1}{120\cdot24\cdot60\cdot60 s}\approx=\frac{1}{10^{7}}$. $\gamma_{C}=1\cdot10^{-7}s^{-1}$.

The transition rate from reticulocytes in the bone marrow to reticulocytes in peripheral blood $\gamma_{R}$ was estimated as follows, based on (1). The reticulocyte maturation time in the bone marrow shortens as anemia severity increases (i.e., at lower hematocrit levels), resulting in an earlier release of younger, less mature reticulocytes into circulation. The relation of maturation time in the peripheral blood to the hematocrit is linear. As the hematocrit is reduced to approximately 35%, the maturation time of the circulating reticulocyte increases from 1.0 to 1.5-1.7 days. With more severe anemia the maturation time will increase to 1.7-2.0 days at a hematocrit of 20-30% and to as much as 2.3-2.5 days at a hematocrit below 20% (2). Linear fit to those 4 points gave the following equation: $\gamma_{R}=\frac{1}{time in bone marrow}=[0.065\cdot Hematocrit + 0.675]^{-1}days^{-1}=\frac{1}{m_{R} C + n_{R}}$ . Converting hematocrit to RBCs and days to seconds, $m_{R}=10^{-8} (cells\cdot s)^{-1}$ and $n_{R}=5.8\times10^{4} s^{-1}$

The maturation rate from CFU-E progenitor cells to reticulocytes was estimated at 2.5 days, based on (3), hence $\tau_{R}=2.16\cdot10^{5} s$.

The degradation rate of EPO by the progenitor cells was estimated as follows, based on (4,5). In steady state, the synthesis rate is equal to the total removal rate.  Denote the removal rate per EPO concentration by $\lambda_{E}$. Hence, ${E_{st}\cdot\lambda}_{E}=4\cdot10^{-4} UL^{-1}s^{-1}$. Substituting $E_{st}=10U/L$, we get $\lambda_{E}=4\cdot10^{-5} s^{-1}$. For a mechanism of endocytosis $\gamma_{E}=\lambda_{E}/H_{st}$. Substituting $H_{st}=6.51\cdot10^{12}U/L$ (the steady state value of the model), we get $\gamma_{E}=6.14\cdot10^{-18}(cells\cdot s)^{-1}$.

The maximal EPO production rate $\sigma_{E}$ and the negative inverse of the loglinear slope of EPO versus RBC, D, were estimated as follows, based on (6–11). In steady state $\sigma_{E}= \lambda_{E}\cdot E_{max}$. Substituting $E_{max}=886 U/L$  (taken from the log-linear fit of EPO versus hemoglobin) and $\lambda_{E}=4\cdot10^{-5} s^{-1}$, we get $\sigma_{E}=\lambda_{E}\cdot886U/L= 0.0354UL^{-1}s^{-1}$.

This value D can be deduced from the slope of the linear line describing log EPO as a function of hemoglobin: $slope=-1/D\to D =-\frac{1}{0.3161}=-3.16 g/dL$. For 5L blood volume, $D =-158.17 g (Hb)$. One RBC contains 0.03 ng of hemoglobin, therefore, 1 gram of Hb is equivalent to $1/3\cdot10^{11}$ RBC (12). Hence, $D =-158.17\cdot\frac{10^{11}}{3}RBC=-5.27\cdot10^{12}RBC.$

The limiting rate in the proliferation term $a_{max}$ was estimated as follows, based on (13). In their study, 195 colonies were observed at saturation, with each colony consisting of 8–49 cells. We used the geometric mean of this range (19.8) to approximate the average number of cells per colony. The total number of cells can be expressed as $cells(t) = cells(0)\cdot exp(a_{max}\cdot t)$. The experiment began with 250 cells, yielding a ratio of 15.4 between the initial and maximal cell numbers. Taking the logarithm gave $a_{max}\cdot t =2.7$. The cells were grown for 6 days, so $a_{max}\cdot6 days =2.7$ $a_{max} =\frac{2.7}{6*24*3600}= 5.3\cdot10^{-6}$.

The Michaelis constant in the proliferation rate term $K_{a}$ was estimated as follows, based on (13). The number of cells at half $a_{max}$ is given by $cells(t) = cells(0)\cdot exp({(a}_{max}/2)\cdot t)$. The authors reported $982.4$ cells, so $982.4=250 cells \cdot exp(0.29days^{-1}\cdot6 days )$. Convert back the cells to colonies, we get 49.6 colonies at half $a_{max}$. In Fig 3 of their paper, Sawada et al. show colony number as a function of EPO concentration. At 49.6 colonies, the EPO concentration was 0.03 U/ml, giving $K_{a}=30 mU/ml$.

The maximal number of CFU-E cells that the bone marrow can sustain, H_max_, was estimated as follows, based on (14). In mice, the peak CFU-E count following severe hemorrhage (60–75% blood volume loss) has been reported to reach approximately 275 × 10³ cells, compared with 84.5 × 10³ under normal conditions. The ratio of active bone marrow mass between mice and humans is estimated to be 1:5,000–1:10,000. Accordingly, the carrying capacity in humans can be approximated as 2.75 × 10⁹ CFU-E cells. Each CFU-E contains 8–49 progenitor cells (geometric mean: 19.2), which divide 3–5 times to form proerythroblasts (15) that subsequently undergo ~4 divisions (16) to yield reticulocytes. In total, each CFU-E gives rise to roughly 10³ mature red blood cells. In our simplified model, each CFU-E is represented as producing a single red blood cell; therefore, we used a scaled value of H_max_ = 1 × 10¹³ to account for all intermediate divisions.

**Bibliography**

1. Prchal JT, Thiagarajan P. Erythropoiesis and Red Cell Turnover. In: Kaushansky K, Prchal JT, Burns LJ, Lichtman MA, Levi M, Linch DC, editors. Williams Hematology, 10e [Internet]. New York, NY: McGraw-Hill Education; 2021. Available from: hemonc.mhmedical.com/content.aspx?aid=1178739323

2. Hillman RS. Characteristics of marrow production and reticulocyte maturation in normal man in response to anemia. J Clin Invest. 1969 Mar 1;48(3):443–53.

3. Hattangadi SM, Wong P, Zhang L, Flygare J, Lodish HF. From stem cell to red cell: regulation of erythropoiesis at multiple levels by multiple proteins, RNAs, and chromatin modifications. Blood. 2011 Dec 8;118(24):6258–68.

4. Bélair J, Mackey MC, Mahaffy JM. Age-structured and two-delay models for erythropoiesis. Math Biosci. 1995;128(1–2):317–46.

5. Jelkmann W. Erythropoietin: structure, control of production, and function. Physiol Rev. 1992 Apr 1;72(2):449–89.

6. Erslev AJ, Caro J, Miller O, Silver R. Plasma erythropoietin in health and disease. Ann Clin Lab Sci. 1980;10(3):250–7.

7. Artunc F, Risler T. Serum erythropoietin concentrations and responses to anaemia in patients with or without chronic kidney disease. Nephrol Dial Transplant. 2007;22(10):2900–8.

8. Bergamaschi G, Markopoulos K, Albertini R, Di Sabatino A, Biagi F, Ciccocioppo R, et al. Anemia of chronic disease and defective erythropoietin production in patients with celiac disease. Haematologica. 2008;93(12):1785–91.

9. Schrezenmeier H, Noé G, Raghavachar A, Rich IN, Heimpel H, Kubanek B. Serum erythropoietin and serum transferrin receptor levels in aplastic anaemia. Br J Haematol. 1994 Oct;88(2):286–94.

10. Schreiber S, Howaldt S, Schnoor M, Nikolaus S, Bauditz J, Gasché C, et al. Recombinant Erythropoietin for the Treatment of Anemia in Inflammatory Bowel Disease. N Engl J Med. 1996;334(10):619–24.

11. Wallner SF, Kurnick JE, Vautrin RM, White MJ, Chapman RG, Ward HP. Levels of erythropoietin in patients with the anemias of chronic diseases and liver failure. Am J Hematol. 1977;3:37–44.

12. Naeim F, Rao PN, Song SX, Grod WW. Disorders of red blood cells—Anemias. Atlas Hematop. 2013;675–704.

13. Sawada K, Krantz SB, Kans JS, Dessypris EN, Sawyer S, Glick AD, et al. Purification of human erythroid colony-forming units and demonstration of specific binding of erythropoietin. J Clin Invest. 1987 Aug 1;80(2):357–66.

14. Iscove NN. THE ROLE OF ERYTHROPOIETIN IN REGULATION OF POPULATION SIZE AND CELL CYCLING OF EARLY AND LATE ERYTHROID PRECURSORS IN MOUSE BONE MARROW. Cell Prolif. 1977 July;10(4):323–34.

15. Lodish H, Flygare J, Chou S. From stem cell to erythroblast: Regulation of red cell production at multiple levels by multiple hormones. IUBMB Life. 2010;62(7):492–6.

16. Hu J, Liu J, Xue F, Halverson G, Reid M, Guo A, et al. Isolation and functional characterization of human erythroblasts at distinct stages: implications for understanding of normal and disordered erythropoiesis in vivo. Blood. 2013 Apr 18;121(16):3246–53.
