# Supplementary material for: A minimal mathematical model of red blood cell homeostasis in anemia
Source: PLoS Comput Biol. 2026 Mar 24;22(3):e1014111. doi: 10.1371/journal.pcbi.1014111 (PMC13048500; doi:10.1371/journal.pcbi.1014111)
Supplement: S2 File — This file describes how the circulating reticulocyte percentage can be inferred from the model’s marrow reticulocyte pool R(t) by decomposing the RBC compartment into circulating reticulocytes and mature erythrocytes, using clinically derived transition rates based on the reticulocyte maturation correction framework. (DOCX) [file pcbi.1014111.s002.docx]

**A minimal mathematical model of red blood cell homeostasis in anemia**

**Supporting information – S2 File**

Herut Dor^1,*^*,* Uri Alon^2,**^

^1^Gray Faculty of Medical and Health Sciences, Tel Aviv University, Tel Aviv, Israel

^2^Department of Molecular Cell Biology, Weizmann Institute of Science, Rehovot, Israel

*Corresponding author

E-mail:[herutdor@gmail.com](mailto:herutdor@gmail.com) (HD)

**Corresponding author

E-mail:[uri.alon@weizmann.ac.il](mailto:uri.alon@weizmann.ac.il) (UA)

**S2 File: Estimation of the circulating reticulocyte percentage from model variables**

The model variable R(t) represents the marrow reticulocyte pool. To relate R(t) to the circulating reticulocyte percentage measured in routine clinical assays, we decompose the original RBC compartment equation (Eq. 3) into two sub-compartments: circulating reticulocytes in the peripheral blood (Rb) and mature erythrocytes (C):

$$\frac{dRb}{dt}=\gamma_{R}\cdot R-\gamma_{Rb}\cdot Rb$$

$$\frac{dC}{dt}=\gamma_{Rb}\cdot Rb-\gamma_{C}\cdot C$$

Here, γ_R_ is the transition rate from bone marrow reticulocytes to peripheral blood (as defined in the main model), and γ_Rb_ is the maturation rate from circulating reticulocytes to mature erythrocytes.

Under normal conditions, reticulocytes undergo their final 3-4 days of maturation in the bone marrow before being released into the peripheral circulation, where they mature into erythrocytes over approximately 1 day (1,2). The total reticulocyte lifespan — from enucleation to full maturation — is thus approximately 4.5 days (≈ 3.9 × 10⁵ s). In anemia, erythropoietic stress causes premature release of less mature ("shift") reticulocytes, shortening the bone marrow transit time and correspondingly extending the peripheral maturation time (3). This phenomenon is well established in clinical practice and forms the basis of the reticulocyte production index (RPI), a standard correction applied to reticulocyte counts to account for the prolonged circulation time of shift reticulocytes (3).

The maturation correction (3) specifies the peripheral blood reticulocyte maturation time as a function of hematocrit: 1.0 day at hematocrit 36–45%, 1.5 days at 26–35%, 2.0 days at 16–25%, and 2.5 days at hematocrit ≤15%. The peripheral blood maturation time can therefore be expressed as:

$$\frac{1}{\gamma_{Rb}}=\tau_{total}-\frac{1}{\gamma_{R}}$$

where τ_total_ ≈ 3.9 × 10⁵ s is the total reticulocyte lifespan and 1/γ_R_ is the bone marrow residence time.

For normal hematocrit (≈ 45%), the bone marrow transit time is 1/γ_R_ ≈ 3 days (2.6 × 10⁵ s) (4), yielding a peripheral blood maturation time of approximately 1 day (8.6 × 10^4^ s) (4) and thus γ_Rb_ ≈ 1.1 × 10⁻^5^ s⁻¹. For the RBC degradation rate in this calculation, we used γ_C_ = 1.16 ×10^-7^ s^-1^(4), which differs slightly from the 120-day value used in the main model because this is the value used to calculate the reticulocytes index.

At steady state, the circulating reticulocyte count (Rb_st_) is:

$$Rb_{st}=R_{st}\cdot\frac{\gamma_{R}}{\gamma_{Rb}}$$

and the reticulocyte percentage is:

$$\%Rb_{st}=\frac{Rb_{st}}{C_{st}}=\frac{R_{st}}{C_{st}}\cdot\frac{\gamma_{R}}{\gamma_{Rb}}$$

We note that these rates represent approximate population-level estimates rather than precisely measured individual quantities.

**Bibliography**

1. Prchal JT, Thiagarajan P. Erythropoiesis and Red Cell Turnover. In: Kaushansky K, Prchal JT, Burns LJ, Lichtman MA, Levi M, Linch DC, editors. Williams Hematology, 10e [Internet]. New York, NY: McGraw-Hill Education; 2021. Available from: hemonc.mhmedical.com/content.aspx?aid=1178739323

2. Hillman RS. Characteristics of marrow production and reticulocyte maturation in normal man in response to anemia. J Clin Invest. 1969 Mar 1;48(3):443–53. doi:10.1172/JCI106001 PubMed PMID: 5773082.

3. Hillman RS, Finch CA. Red Cell Manual. Philadelphia: F A Davis Co; 1996. 190 p.

4. Longo D, Fauci AS, Kasper DL, Hauser S, Jameson JL, Loscalzo J, et al. Harrison’s Principles of Internal Medicine, Twenty-Second Edition. New York: McGraw Hill / Medical; 2025.
